# The Mitochondrial Chaperone Protein TRAP1 Mitigates α-Synuclein Toxicity

**DOI:** 10.1371/journal.pgen.1002488

**Published:** 2012-02-02

**Authors:** Erin K. Butler, Aaron Voigt, A. Kathrin Lutz, Jane P. Toegel, Ellen Gerhardt, Peter Karsten, Björn Falkenburger, Andrea Reinartz, Konstanze F. Winklhofer, Jörg B. Schulz

**Affiliations:** 1 Department of Neurology, University Medical Center, RWTH Aachen, Aachen, Germany; 2 Department of Neurodegeneration and Restorative Research, Center Molecular Physiology of the Brain (CMPB), Georg-August University Göttingen, Göttingen, Germany; 3 Göttingen Graduate School for Neurosciences and Molecular Biology (GGNB), Göttingen, Germany; 4 Neurobiochemistry, Adolf-Butenandt-Institute, Ludwig Maximilians University, Munich, Germany; 5 Department of Pathology, University Medical Center, RWTH Aachen, Aachen, Germany; 6 German Center for Neurodegenerative Diseases (DZNE), Munich, Germany; 7 Jülich-Aachen Research Alliance (JARA) Brain, Jülich/Aachen, Germany; University of Minnesota, United States of America

## Abstract

Overexpression or mutation of α-Synuclein is associated with protein aggregation and interferes with a number of cellular processes, including mitochondrial integrity and function. We used a whole-genome screen in the fruit fly *Drosophila melanogaster* to search for novel genetic modifiers of human [A53T]α-Synuclein–induced neurotoxicity. Decreased expression of the mitochondrial chaperone protein tumor necrosis factor receptor associated protein-1 (TRAP1) was found to enhance age-dependent loss of fly head dopamine (DA) and DA neuron number resulting from [A53T]α-Synuclein expression. In addition, decreased TRAP1 expression in [A53T]α-Synuclein–expressing flies resulted in enhanced loss of climbing ability and sensitivity to oxidative stress. Overexpression of human TRAP1 was able to rescue these phenotypes. Similarly, human TRAP1 overexpression in rat primary cortical neurons rescued [A53T]α-Synuclein–induced sensitivity to rotenone treatment. In human (non)neuronal cell lines, small interfering RNA directed against TRAP1 enhanced [A53T]α-Synuclein–induced sensitivity to oxidative stress treatment. [A53T]α-Synuclein directly interfered with mitochondrial function, as its expression reduced Complex I activity in HEK293 cells. These effects were blocked by TRAP1 overexpression. Moreover, TRAP1 was able to prevent alteration in mitochondrial morphology caused by [A53T]α-Synuclein overexpression in human SH-SY5Y cells. These results indicate that [A53T]α-Synuclein toxicity is intimately connected to mitochondrial dysfunction and that toxicity reduction in fly and rat primary neurons and human cell lines can be achieved using overexpression of the mitochondrial chaperone TRAP1. Interestingly, TRAP1 has previously been shown to be phosphorylated by the serine/threonine kinase PINK1, thus providing a potential link of PINK1 via TRAP1 to α-Synuclein.

## Author Summary

Parkinson's disease (PD) is a progressive neurodegenerative disorder, pathologically characterized by loss of dopaminergic neurons in the substantia nigra pars compacta brain region. Mutations in α-Synuclein or gene duplication or triplication result in autosomal-dominant inherited PD. Indeed, aggregated and insoluble α-Synuclein is found in Lewy bodies, a pathological hallmark common to both sporadic and hereditary forms of PD. In order to better define α-Synuclein's pathogenic mechanism, we first used a fly genetic screen to search for novel genetic modifiers of mutant human [A53T]α-Synuclein neurotoxicity. We identified the mitochondrial chaperone protein TRAP1 as a novel modifier of the toxicity induced by [A53T]α-Synuclein. [A53T]α-Synuclein–induced toxicity was enhanced when TRAP1 expression was decreased, while overexpression of human TRAP1 (hTRAP1) provided a rescue. Cell culture experiments further demonstrated that [A53T]α-Synuclein directly interferes with a number of mitochondrial functions, including Complex I ATP production, mitochondrial fragmentation, and sensitivity to oxidative stress. These effects could be blocked by TRAP1 overexpression. As mitochondrial dysfunction has previously been linked to mutations in several other genes associated with genetic PD, these data provide further evidence of a common mitochondrial-centric mechanism of PD pathogenesis.

## Introduction

Parkinson's disease (PD) is the second most prevalent neurodegenerative disease behind Alzheimer's disease (AD), with an incidence rate of approximately 110–300 per 100,000 persons above the age of 50 [1]. The movement disorder is characterized by the selective death of dopaminergic neurons in the substantia nigra pars compacta (SNc) [2]. Death of SNc neurons results in a reduction of dopamine (DA) levels within their key efferent target, the striatum [3]. Mitochondrial Complex I activity deficit and evidence of enhanced oxidative stress within affected brain regions are also observed in PD [4]–[6]. Age and pesticide/herbicide exposure are the most important disease risk factors [7]–[9]. Importantly, there is no clinical therapy available that has been shown to slow or reverse PD.

While the majority of PD is diagnosed as idiopathic, 5–10% of cases are attributable to familial forms of PD [10]. Although genetic PD represents only a small percentage of patients, mutations in these genes point to underlying biochemical pathways that could also be relevant to sporadic PD patients. Three missense mutations in the small pre-synaptic protein α-Synuclein (SNCA/PARK1/4; GenBank ID 6622) have been shown to result in autosomal-dominant PD. A critical effect of protein dose on pathology is implicated by disease-causing gene duplication and triplication [11]–[14]. α-Synuclein is also a major protein component of the Lewy Bodies (LB), the key histologic feature of dopaminergic and non-dopaminergic neurons found in PD patients [15]. Thus, α-Synuclein is strongly suggested to be a causal factor in PD pathogenesis.

Human α-Synuclein mutation or overexpression results in cytotoxicity, with [A53T]α-Synuclein being the most toxic variant known. Direct cell loss can be induced in both *in vitro* and *in vivo* models of yeast, *C. elegans*, *Drosophila*, rat, mouse, and non-human primate [16]–[23]. The formation of α-Synuclein oligomers from their native unfolded state is linked to cell membrane damage and results in dysfunction of multiple cell systems such as the ubiquitin proteasome system, the endoplasmic reticulum and lysosomes [24]–[31]. Recent data also suggests that α-Synuclein plays a role in modulating both mitochondrial function and damage. α-Synuclein-overexpressing cells exhibit multiple markers of mitochondrial dysfunction, including increased protein oxidation, increased ROS production, loss of mitochondrial membrane potential and reduced Complex I activity [32]–[38]. Several groups have demonstrated that α-Synuclein's entry in mitochondria is mediated via an N-terminal mitochondrial targeting sequence, with localization at the inner membrane [37], [38]. Moreover, PD patient brain histology shows α-Synuclein accumulation within mitochondria of the SNc and striatum, a feature absent in control brains [38]. Mitochondrial dysfunction associated with adenosine triphosphate (ATP) depletion and electron transport chain (ETC) defects reduces the cell's ability to handle oxidative protein damage and cellular tasks, suggesting a possible reason for cell death.

In PD patient brains, early DA reduction indicates the withdrawal of SNc striatal projections, finally resulting in DA neuron loss and PD-related symptoms of rigidity and akinesia. In flies, expression of [A53T]α-Synuclein is accompanied by an age-dependent loss of DA and DA neurons, respectively. Thus, fly head DA levels provide an indirect readout for [A53T]α-Synuclein-induced toxicity. To further investigate the mechanism and identify novel modifiers of α-Synuclein toxicity, we performed a genome-wide genetic screen in *Drosophila*. In this screen, we identified, among other gene products, the mitochondrial chaperone protein TRAP1 (GenBank ID 10131) as a novel modifier of [A53T]α-Synuclein-induced DA loss. TRAP1 has previously been shown to function downstream of the PD-related serine/threonine kinase PINK1 (GenBank ID 65018). PINK1-induced phosphorylation of TRAP1 seems to be necessary for the protein's protective effects against oxidative stress [39]. In our report we further characterize the functional consequences of TRAP1 reduction or overexpression in *Drosophila*, in primary neurons and dopaminergic cell lines and the effects on mitochondrial morphology and function.

## Results

### Expression of Human [A53T]α-Synuclein in Fly Heads and Genetic Screening for Modification of [A53T]α-Synuclein Toxicity

**Figure 1:**
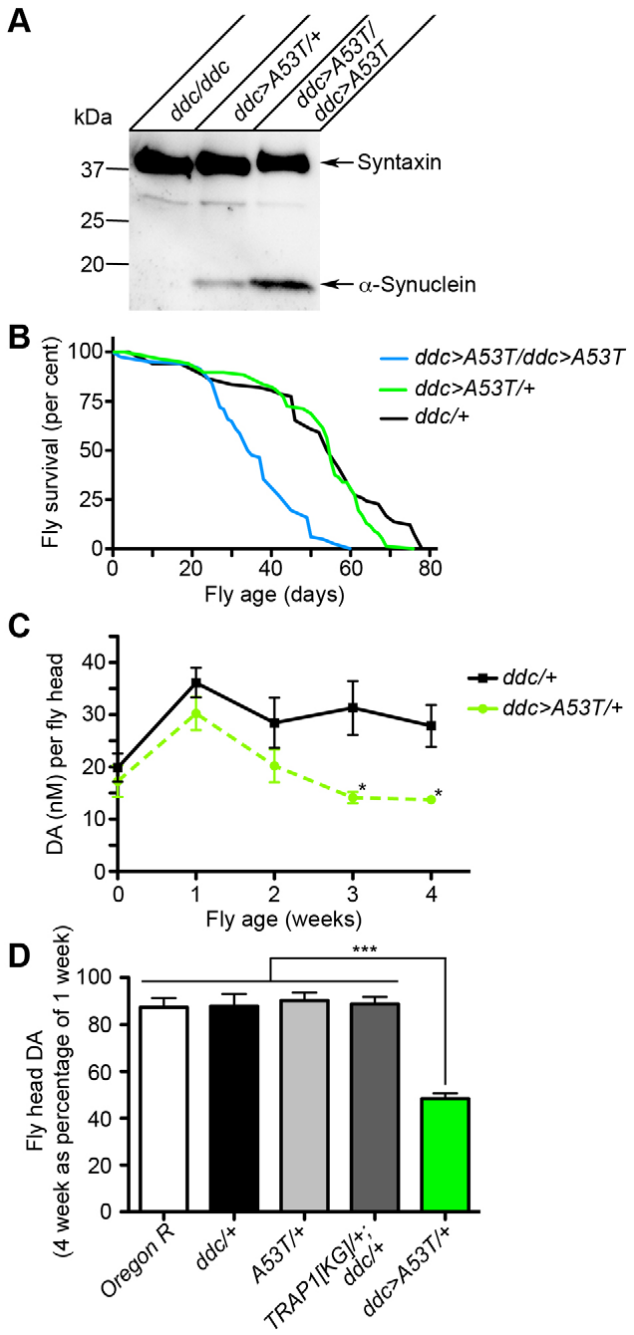
[A53T]α-Synuclein expression in fly heads results in age-dependent loss of DA. (A) Western blot showing abundance of human [A53T]α-Synuclein after aminergic neuron (*ddc-GAL4*)-specific expression in lysates of fly heads. While flies without [A53T]α-Synuclein transgene do not show any detectable signal for [A53T]α-Synuclein in Western blot, an increase of [A53T]α-Synuclein protein levels was observed by increasing the copy number of transgenes. Syntaxin served as a loading control and molecular weight markers are indicated. (B) Whereas *ddc>A53T/+* flies displayed no significant difference in longevity as compared to *ddc/+* flies, flies homozygous for *ddc>A53T* showed a significant decrease (p<0.001, Log rank test). (C) Compared to controls (*ddc/+*), *ddc>A53T*/+ flies showed a significant age-dependent loss of DA at 3 and 4 weeks post eclosion (*p<0.05 vs. control). (D) Only *ddc>A53T* flies showed a significant decrease (***p<0.001) in DA concentration in fly heads at 4 weeks. Comparisons of multiple controls were not significant (4 week values as per cent of 1 week values; ANOVA followed by Newman-Keuls Multiple Comparison Test).

Expression of α-Synuclein in *Drosophila* is established as a useful model of PD [21]. As the fly lacks an α-Synuclein homolog, this model relies on ectopic expression of human α-Synuclein using the *UAS/GAL4* system [40]. We have previously analyzed DA neuron number in aged flies, expressing different mutant variants of α-Synuclein. Compared to controls, wild type α-Synuclein did not cause a decline in DA neuron number. Moreover, locomotion was not impaired in aged wild type α-Synuclein-expressing flies [41]. Based on these results, we chose [A53T]α-Synuclein for our screening. With single copy expression of a *UAS:[A53T]α-Synuclein* transgene (*A53T*) in aminergic neurons (*dopa decarboxylase-GAL4* driver, *ddc-GAL4*) (Figure 1A), no difference to overall fly fitness, as assessed by longevity, was observed (Figure 1B). In contrast, expression of two transgene copies, resulting in higher expression levels (Figure 1A), caused earlier lethality compared to controls (Figure 1B). However, when we measured DA levels of flies expressing one copy of [A53T]α-Synuclein under control of *ddc-GAL4* (*ddc>A53T*), we noticed a significant decrease in DA levels with aging (Figure 1C). Thus, measuring DA levels using high performance liquid chromatography represents a sensitive system to address DA levels in fly heads. After carefully addressing sensitivity, specificity and reproducibility of our readout marker (Figure 1D, Figure S1), we decided to perform a genome-wide screen to identify modifiers of [A53T]α-Synuclein-induced DA loss *in vivo.* Thus, flies with expression of [A53T]α-Synuclein in aminergic neurons were crossbred to fly lines carrying chromosomal deletions (deficiencies), utilizing the “Bloomington Deficiency Kit”. Progeny were screened for changes in DA loss over time (a summary of the screen results is given in Text S1 and Tables S1, S2, S3, S4). Although detailed single gene analysis is still ongoing, we identified a large number of genes coding for proteins involved in mitochondrial function within the candidate deficiencies. Therefore, we additionally cross-referenced our data with results from a genome-wide RNAi-screen, set to identify modulators of mitochondrial function [42]. Common genes were screened for alteration of [A53T]α-Synuclein-toxicity with respect to viability and DA loss.

### TRAP1 Modification of [A53T]α-Synuclein Toxicity in the Fly

**Figure 2:**
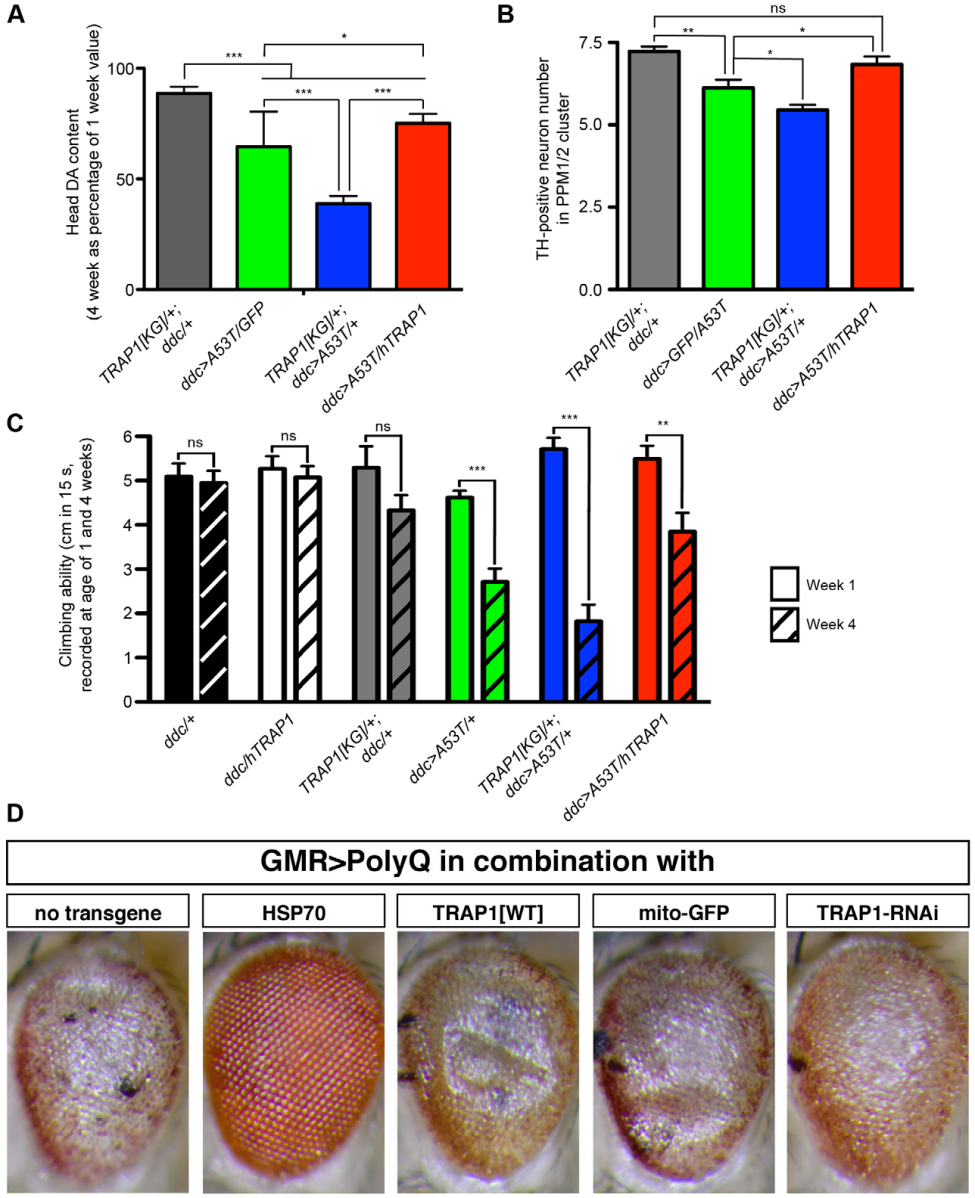
TRAP1 overexpression mitigates detrimental effects induced by neuronal [A53T]α-Synuclein expression. (A) Overexpression of [A53T]α-Synuclein under control of *ddc-GAL4* resulted in reduction of DA in fly heads at 4 weeks, which was potentiated by TRAP1 deficiency (*TRAP1[KG]/+;ddc>A53T/+*), but mitigated by TRAP1 overexpression (*ddc>A53T/hTRAP1*). (B) *ddc>A53T* flies display a reduction of TH-positive neurons, which was potentiated by TRAP1 deficiency, but rescued to control levels by TRAP1 overexpression. (C) In negative geotaxis assays *ddc>A53T* flies displayed a time-dependent decline in locomotion. Reduction of TRAP1 enhanced the inability to climb (although not significant), while overexpression of hTRAP1 provided a significant rescue effect (comparison of *ddc>A53T/+* vs *ddc>A53T/hTRAP1* at 4 weeks: p<0.05). Statistics in (A, B): ANOVA followed by Newman-Keuls Multiple Comparison Test; (C): 2-way ANOVA followed by Bonferroni post-hoc tests. Displayed are biologically relevant comparisons. *p<0.05; **p<0.01; ***p<0.001; ns = not significant. (D) Alterations in TRAP1 levels did not influence PolyQ-induced rough eye phenotypes. Light micrographs of external eye structures show that PolyQ-induced REP was suppressed by parallel expression of HSP70. In contrast, neither overexpression of hTRAP1 or silencing of endogenous TRAP1 by RNAi had an obvious impact on external eye structure. Expression of mitochondrial localized GFP (mito-GFP) served as control.

Among the deficiencies screened, *Df(2R)nap9* caused the greatest enhancement of [A53T]α-Synuclein-induced DA loss of all non-lethal interacting deficiencies. Of the 153 genes deleted by *Df(2R)nap9*, we found TRAP1 reduction to enhance [A53T]α-Synuclein-induced DA loss. The loss-of-function allele *TRAP1[KG06242]* (hereafter referred to as *TRAP1[KG]*, Figure S2) caused a reduction of fly head DA levels similar to those of *Df(2R)nap9* (data not shown). However, *TRAP1[KG]* did not alter DA levels (Figure 1D). Thus, flies with reduced TRAP1 in combination with the *ddc-GAL4* driver (*TRAP1[KG]/+; ddc/+*) served as controls in later analysis. TRAP1 is a mitochondrial chaperone, recently reported as a downstream phosphorylation target of the PD protein PINK1 in rat and human cell lines [39]. As both fly and human TRAP1 share high sequence homology, we generated a UAS-transgenic fly to express human TRAP1 (hTRAP1). Interestingly, overexpression of hTRAP1 in fly heads was able to provide a rescue effect against [A53T]α-Synuclein-induced DA loss (Figure 2A). The effects of TRAP1 on DA levels were also reflected by its effect on tyrosine hydroxylase (TH)-positive neurons. *ddc>A53T* expressing flies showed increased loss of TH-positive neurons if TRAP1 levels were reduced (Figure 2B). In contrast, hTRAP1 overexpression was able to restore [A53T]α-Synuclein-induced loss of TH-positive neurons to control levels. Interestingly, *ddc-*driven overexpression of hTRAP1 did not increase longevity of *ddc>A53T* heterozygous flies (not shown). In PD patients, a reduction in brain DA content is later followed by neuronal decline. The same seems to hold true for flies. Although no reduction in longevity of *ddc>A53T/*+ flies is observed, these flies display a significant reduction in DA content. A more pronounced DA reduction (*ddc>A53T/ddc>A53T*) results in neuronal decline, eventually leading to early death, reflected by a significantly shortened lifespan (Figure 1B). This might explain why TRAP1 will still provide 100% protection against loss of neurons (Figure 2B), even if DA levels have already started to decline (Figure 2A).

PD is clinically defined as a movement disorder. Thus, key to an animal disease model recapitulating this phenotype is loss of locomotor ability. Locomotion in flies is measurable using the negative geotaxis assay. In agreement with previous reports, *ddc>A53T* flies showed an age-related deficit in climbing ability [21], [43]. Notably, *ddc-*driven hTRAP1 expression was able to significantly rescue the locomotion deficit in *ddc>A53T* flies (Figure 2C). Therefore, taken together, these data indicate that the rescue of head DA content is sufficient to restore motor ability.

Our data suggest that TRAP1 protects from toxic effects induced by [A53T]α-Synuclein. However, TRAP1 might also provide protection to any toxic insult. To address this possibility, we examined if TRAP1 provides protection against toxicity induced by the expression of other well-known toxic proteins/peptides.

Eye-specific expression (GMR-GAL4) of either human Tau or SCA3-derived polyglutamine stretches (PolyQ) in the fly eye result in a rough eye phenotype (REP). These REPs are sensitive to genetic modifiers and have successfully been used for screening [44], [45]. Interestingly, overexpression of TRAP1 did not show a rescue of the PolyQ-induced REP. Moreover, silencing of TRAP1 by RNAi did not enhance the REP (Figure 2D). Similar results were obtained with Tau-expressing flies (not shown). A general protective role of TRAP1 in any toxic trigger is therefore unlikely but appears to be specific for α-Synuclein toxicity.

### Modification of [A53T]α-Synuclein Toxicity by TRAP1 in Rat Primary Cortical Neuron Culture

**Figure 3:**
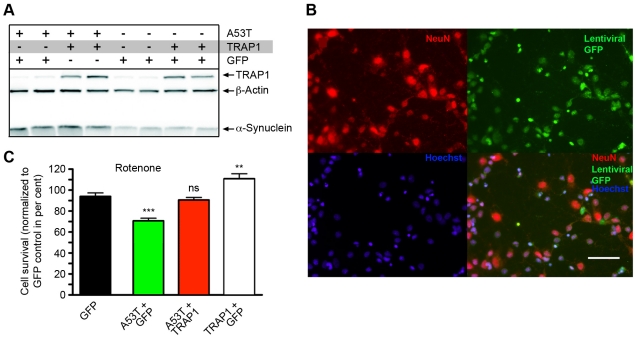
TRAP1 overexpression protects rat cortical neurons from [A53T]α-Synuclein-induced sensitivity to rotenone. (A) Western blot analysis of cells infected with lentivirus promoting either GFP, TRAP1 or [A53T]α-Synuclein expression. For visualization, the blot was probed with either TRAP1- or α-Synuclein-specific antibodies, respectively. ß-Actin was used for normalization. (B) Rat cortical neurons infected with lentivirus promoting GFP expression were stained for neuronal marker NeuN (red) and DNA (Hoechst, blue). A high percentage of cells showed co-localization between GFP and the neuronal marker NeuN, indicative for high infection efficacy. Scale bar indicates 43 µm. (C) Quantification of cell survival after 16 h of rotenone treatment of cells infected with viruses mediating expression of indicated protein. Significant differences compared to control (GFP) are indicated. All other comparisons revealed highly significant differences (p<0.001) in statistical analysis (ANOVA followed by Newman-Keuls Multiple Comparison Test). **p<0.01; ***p<0.001; ns = not significant.

In flies, overexpression of hTRAP1 was able to reduce [A53T]α-Synuclein-induced loss of DA in fly heads and loss of DA neurons, respectively. Thus, to additionally confirm that overexpression of hTRAP1 is able to rescue [A53T]α-Synuclein-induced sensitivity in vertebrate neurons, we used terminally-differentiated rat primary neuron cultures. Lentiviral infection specificity and efficacy in these cells was first verified (Figure 3A, 3B). As the neurons did not display robust toxicity upon [A53T]α-Synuclein expression alone, cells were exposed to low doses of the mitochondrial Complex I inhibitor rotenone. Compared to GFP-virus infected cells (control), co-expression of [A53T]α-Synuclein significantly enhanced cell loss (Figure 3C). In agreement with fly data, coincident overexpression of TRAP1 restored survival to control values. Interestingly, expression of TRAP1 alone enhanced survival beyond that of control cells, indicative of a protective effect of TRAP1 on neurons independent of effects on [A53T]α-Synuclein-induced toxicity.

### Modification of [A53T]α-Synuclein Toxicity by TRAP1 in Human HEK293 Cell Culture

To study the functional role of TRAP1, we used both TRAP1 overexpression and specific knockdown by small interfering RNA (siRNA) in human embryonic kidney cells-293 (HEK293) cells. Two different siRNAs directed against TRAP1 were first compared for efficacy. Both were able to reduce endogenous TRAP1 expression in HEK293 cells (Figure S3). The most efficient siRNA was used for all further investigations.

**Figure 4:**
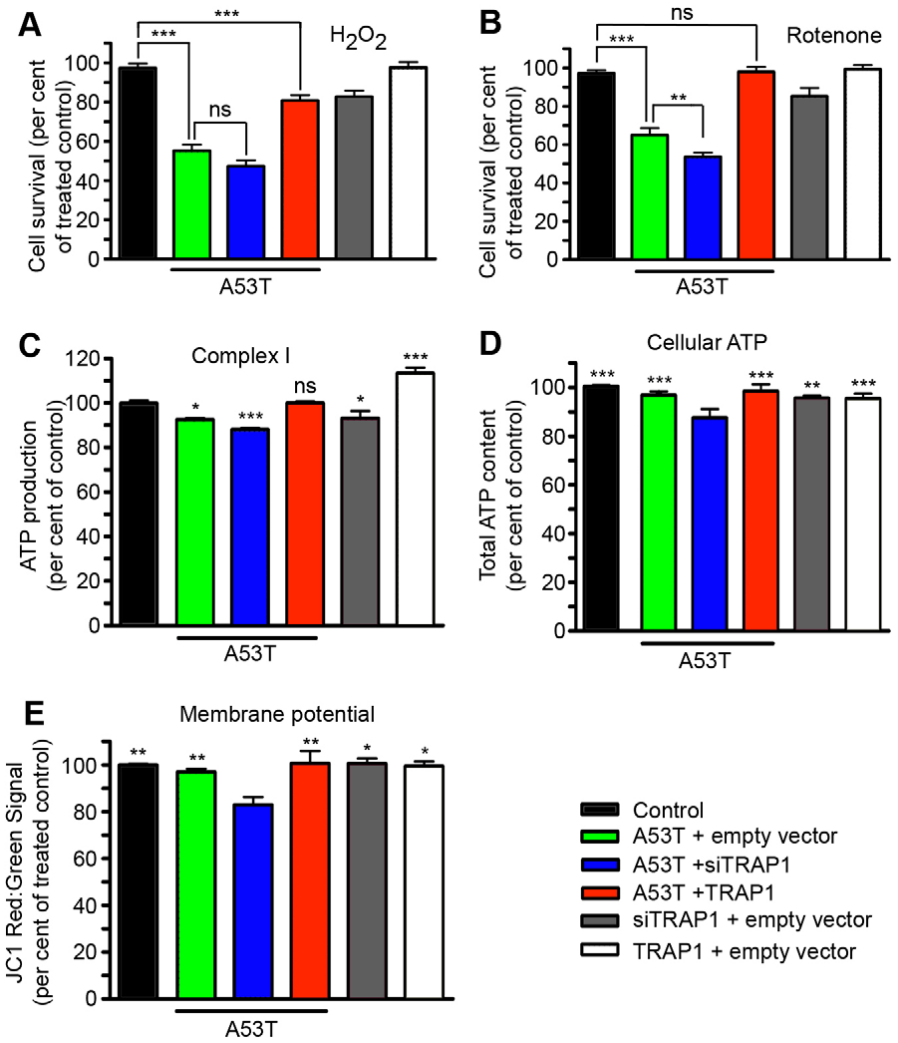
Alterations in TRAP1 levels influence [A53T]α-Synuclein-induced sensitivity to oxidative stress and mitochondrial effects in HEK293 cells. HEK293 cells were transfected with plasmids promoting [A53T]α-Synuclein or TRAP1 expression. Empty vector transfection served as control. In addition, RNAi-mediated silencing of endogenous TRAP1 was induced (siTRAP1). Cells transfected with indicated plasmid combinations were treated with (A) hydrogen peroxide (100 µm) or (B) rotenone (200 µM) to induce oxidative stress. Cell numbers were analyzed to monitor survival. (C–E) HEK293 without oxidative stress treatment overexpressing the indicated proteins, or with RNAi-mediated silencing of TRAP1 were analyzed for (C) ATP production via Complex I, (D) total ATP content, and (E) mitochondrial membrane potential. Statistical analysis of displayed bar graphs was performed using ANOVA followed by Newman-Keuls Multiple Comparison Test. (A, B) Biologically relevant comparisons are indicated in bar graphs. (C) Differences compared to control are indicated. (A, B, C) A detailed summary of all comparisons is summarized in Figure S8. (D, E) Only cells with [A53T]α-Synuclein expression and TRAP1 reduction displayed significant differences in statistical analysis as indicated in graph. All other comparisons were not significant. *p<0.05; **p<0.01; ***p<0.001; ns = not significant.

To confirm whether treatment of HEK293 cells mimicked the *in vivo* fly and *in vitro* rat neuron data concerning TRAP1 and stress sensitivity, HEK293 cells were treated overnight with a low dose of either hydrogen peroxide (Figure 4A) or the Complex I inhibitor rotenone (Figure 4B). [A53T]α-Synuclein expression enhanced cell sensitivity to both stressors. Reduction in TRAP1 expression further reduced survival in the presence of [A53T]α-Synuclein. For both, rotenone and hydrogen peroxide treatment, overexpression of TRAP1 in the context of [A53T]α-Synuclein expression attenuated the decrease in cell survival. The magnitude of the rescue effect was greatest when cells were exposed to rotenone (Figure 4A, 4B). In cells without [A53T]α-Synuclein expression, reduction of TRAP1 also caused stress sensitization. These data corroborate the reported function of TRAP1 as a protective mitochondrial chaperone [46]–[48].

Previous reports have indicated that [A53T]α-Synuclein may interfere with mitochondrial respiration, in particular with Complex I function [34]. Given the noted rescue effect of TRAP1 on rotenone-treated cells with or without [A53T]α-Synuclein expression, we hypothesized that the TRAP1 effect on [A53T]α-Synuclein may in part be related to altered ETC function. Thus, ATP production via Complex I was assayed in cells without oxidative stress, to evaluate the general effects of [A53T]α-Synuclein on ETC in combination with altered TRAP1 levels. Expression of [A53T]α-Synuclein reduced Complex I activity in HEK293 cells (Figure 4C). TRAP1-silencing enhanced this reduction, while TRAP1 overexpression rescued the [A53T]α-Synuclein-induced defect. In light of the defects observed in [A53T]α-Synuclein-induced Complex I ATP production (Figure 4C), total ATP levels in the cell were also investigated. Only cells expressing [A53T]α-Synuclein in combination with siTRAP1 showed a reduction of total ATP levels (Figure 4D). Although [A53T]α-Synuclein alone significantly reduced Complex I activity, overall ATP levels were unchanged.

Loss of mitochondrial membrane potential predisposes cells to apoptosis. [A53T]α-Synuclein has been suggested to adopt an alpha-helical conformation that could perforate membranes. At the same time, TRAP1 protection against apoptosis has been suggested to act via inhibition of opening mitochondrial permeability transition pore (PTP) [49]. The mitochondrial membrane potential is thought to indirectly reflect the state of the PTP. Cells were thus assessed for mitochondrial membrane potential using the mitochondrial membrane dye, JC-1. Only cells expressing [A53T]α-Synuclein in combination with siTRAP1 showed a loss of mitochondrial membrane potential (Figure 4E).

Finally, to exclude the possibility that altered Complex I ATP production might be due to varying quantites of mitochondria within the cells, instead of a functional deficit in the ETC, cell samples were probed for two mitochondrial proteins, VDAC1 and COX4. No major differences were observed for expression of VDAC1 and COX4 (Figure S4A). This suggests the detected decrease in Complex I ATP production resulted from a functional ETC deficit. JC-1 is an excellent dye to measure mitochondrial membrane potential and because of the color switch following depolarization, it makes it easy to normalize to cell density. However, JC-1 has been superseded by other dyes, like TMRM, with respect to the potential artifact of local concentration changes. With regard to this potential problem, we repeated mitochondrial membrane potential measurements using TMRM. In addition, we wanted to exclude potential off-target effects by siRNA treatment. Therefore, we generated HEK293 cells with stable expression of shTRAP1 constructs resulting in a roughly 90% loss of TRAP1 protein levels (Figure S5A). In stable TRAP1-silenced cells, a significant reduction in membrane potential was observed after [A53T]α-Synuclein expression. This effect was absent in cells expressing scrambled shRNA, again indicating that TRAP1-silencing in combination with [A53T]α-Synuclein expression causes opening of mitochondrial PTP (Figure S5B).

### Effect of TRAP1 Mutation on [A53T]α-Synuclein–Induced Toxicity

**Figure 5:**
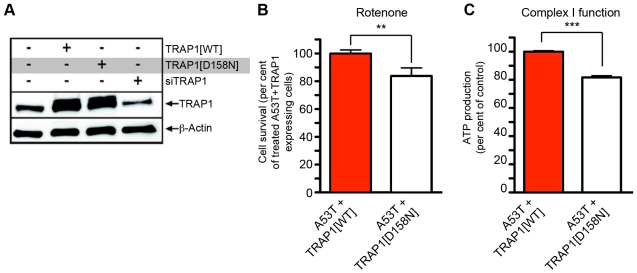
Effect of TRAP1 mutation on modification of [A53T]α-Synuclein toxicity. (A) Western blot analysis of HEK293 lysates transfected with indicated constructs showed similar expression levels of TRAP1[WT] and TRAP1[D158N] and a reduction of endogenous TRAP1 by siTRAP1. Blot was probed with TRAP1-specific antibody. ß-Actin served as loading control. (B, C) Effect of TRAP1[WT] and TRAP1[D158N] on [A53T]α-Synuclein-induced effects in HEK293 cells. (B) Effect of TRAP1[D158N] on [A53T]α-Synuclein-induced toxicity. Cell survival after rotenone (200 µM) treatment was monitored. Compared to TRAP1[WT], cells expressing TRAP1[D158N] displayed a significant reduction in survival (t-test, **p<0.01). (C) Assessment of ATP production via Complex I in unstressed cells with [A53T]α-Synuclein expression revealed a significant reduction of ATP levels in TRAP1[D158N] versus TRAP1[WT] expressing cells (t-test, ***p<0.001).

The human TRAP1 ATPase domain shares high homology with both other HSP90 proteins and TRAP1 orthologs found in other species (Figure S6). Recently, the ATPase domain of yeast HSP90 has been shown to be required for its HSP90 function. The mutation of a specific amino acid within the ATPase domain was sufficient to inhibit ATP binding [50]. This amino acid is highly conserved in both HSP90 and TRAP1 proteins (Figure S6). We therefore exchanged the aspartic acid at position 158 for asparagine (TRAP1[D158N]), creating a putative non-functional ATPase domain. Introducing the D158N mutation did not interfere with TRAP1 protein turnover, as expression in HEK293 cells resulted in similar abundance of TRAP1[D158N] and TRAP1[WT] proteins (Figure 5A).

Next, we asked if TRAP1[D158N] is as effective as TRAP1[WT] in protecting [A53T]α-Synuclein-expressing cells from oxidative stress. Cells overexpressing [A53T]α-Synuclein treated overnight with rotenone displayed a robust reduction in cell survival, which was rescued by TRAP1[WT] overexpression (Figure 4B). In contrast, overexpression of TRAP1[D158N] was less effective (Figure 5B). Similar results were observed when we tested ATP production by Complex I. In the context of [A53T]α-Synuclein expression without oxidative stress, TRAP1[WT] rescued [A53T]α-Synuclein-induced decrease in Complex I ATP production (Figure 5C), while TRAP1[D158N] showed significantly lower degree of rescue ability. Finally, cell lysates were again analyzed for abundance of the mitochondrial proteins VDAC1 and COX4. No changes in VDAC1 or COX4 protein levels were observed in cell lysates expressing either TRAP1[WT] or mutant TRAP1[D158N] (Figure S4B). These data thus indicate that mutant TRAP1 expression does not alter the overall mitochondrial content, arguing in favor of a functional ETC Complex deficit, rather than a deficit due to diminished numbers of mitochondrial/ETC components in the cell.

**Figure 6:**
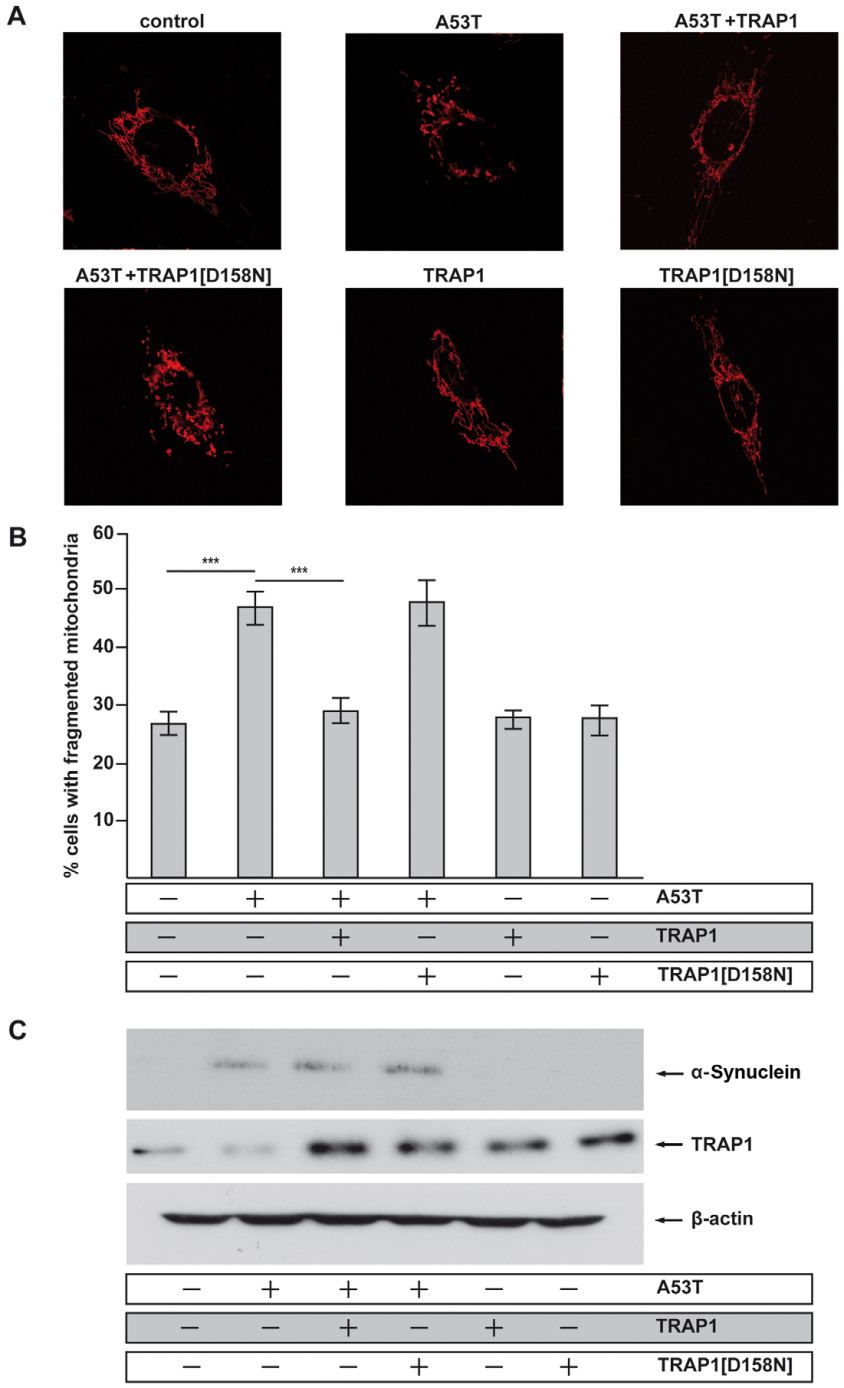
Inhibition of mitochondrial fusion by [A53T]α-Synuclein is rescued by TRAP1. SH-SY5Y cells were co-transfected with the indicated constructs and mito-DsRed to visualize mitochondria. The mitochondrial morphology of transfected cells was analyzed by fluorescence microscopy. (A) Confocal images of representative cells displaying either an intact tubular mitochondrial network (control) or a fragmentation of this network (A53T). Co-expression of TRAP1[WT] prevented [A53T]α-Synuclein-induced mitochondrial fragmentation, whereas the TRAP1[D158N] mutant did not show rescue activity. Overexpression of either wild type or mutant TRAP1 alone did not influence mitochondrial morphology under steady state conditions. (B) For quantification, the mitochondrial morphology of at least 300 transfected cells per coverslip was determined in a blinded manner. Quantifications were based on triplicates of at least three independent experiments. Shown is the percentage of cells with fragmented mitochondria. (C) Expression levels of [A53T]α-Synuclein and TRAP1 were analyzed by Western blotting. β-Actin served as a loading control. ***p<0.001 (ANOVA).

**Figure 7:**
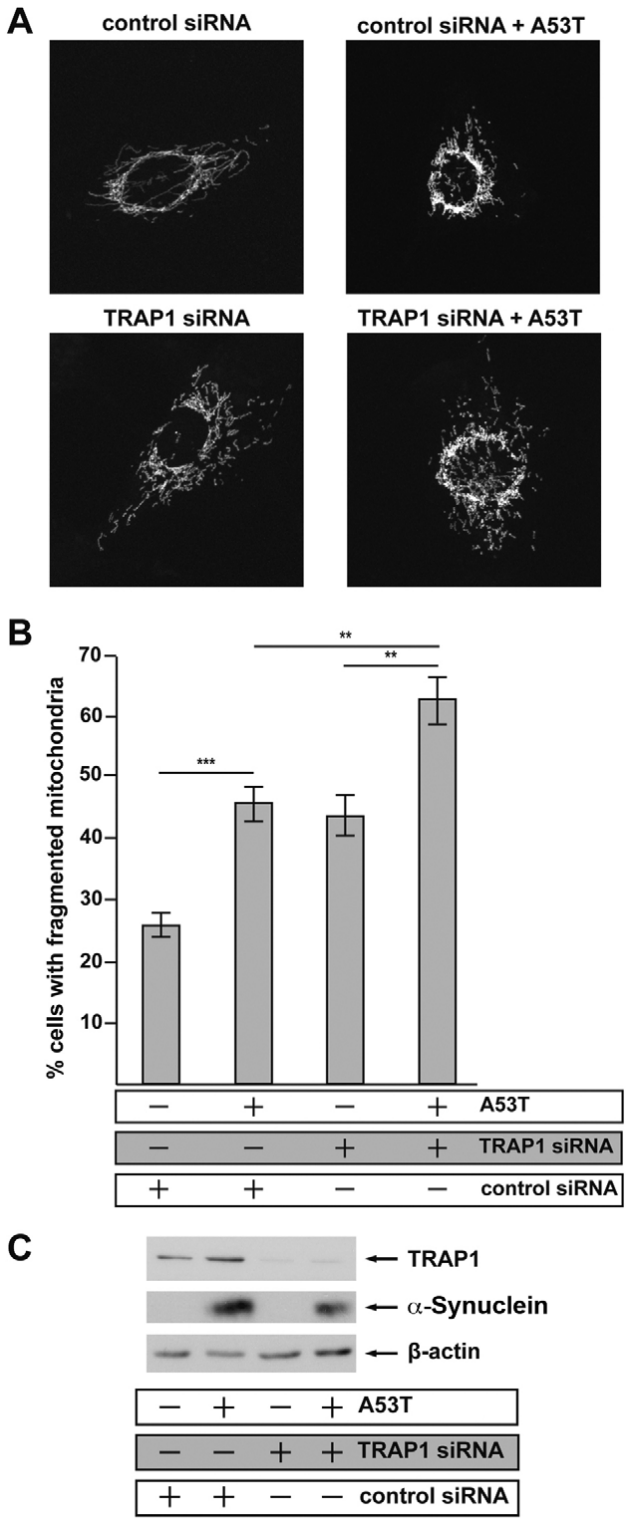
Transient siRNA-mediated knockdown of TRAP1 increases [A53T]α-Synuclein-induced mitochondrial fragmentation. SH-SY5Y cells were co-transfected with the siRNAs and plasmids indicated. Mitochondria were visualized by DsRed targeted to mitochondria (mito-DsRed). (A) Confocal images taken of representative cells displaying either an intact tubular mitochondrial network (control siRNA) or a fragmentation of the network (control siRNA+A53T). Transient knockdown of TRAP1 causes mitochondrial fragmentation itself (TRAP1 siRNA), additional co-expression of [A53T]α-Synuclein aggravated this phenotype and led to an overall increase in cells showing a fragmented mitochondrial network (TRAP1 siRNA+A53T). (B) For quantification, at least 300 transfected cells per coverslip were analyzed. The mitochondrial morphology was determined in a blinded manner. Quantifications are based on triplicates of three independent experiments. (C) Expression levels of [A53T]α-Synuclein and TRAP1 were analyzed by Western Blotting. β-Actin was used as a loading control. **p<0.01; ***p<0.001 (ANOVA).

Recent data show that α-Synuclein impairs mitochondrial fusion, leading to fragmented mitochondria. Interestingly, the α-Synuclein-induced mitochondrial fragmentation can be attenuated by co-expression of PINK1, Parkin and DJ-1, but not by PD-linked mutant variants of these proteins [51]. Therefore, we sought to determine if TRAP1 is also able of attenuating α-Synuclein-induced mitochondrial fragmentation in SH-SY5Y cells. The [A53T]α-Synuclein-induced punctate mitochondrial staining was reversed to a tubular mitochondrial network by TRAP1[WT] co-expression. In contrast, co-expression of TRAP1[D158N] showed no effect (Figure 6A, 6B). The expression of both TRAP1 variants alone had no impact on mitochondrial integrity. Verification of protein expression levels revealed robust α-Synuclein and TRAP1 expression after transfection with respective plasmids (Figure 6C). Thus, the impaired rescue ability of TRAP1[D158N] in comparison to TRAP1[WT] cannot be explained by the lower abundance of TRAP1[D158N] protein. It is rather the consequence of an altered function of the inherent ATPase function of TRAP1[D158N]. In addition, we asked if reduced TRAP1 levels might enhance mitochondrial fragmentation induced by [A53T]α-Synuclein expression. We noticed that TRAP1-silencing increased the number of cells with fragmented mitochondria. Combining TRAP1-silencing with [A53T]α-Synuclein expression enhanced fragmentation of mitochondria even further (Figure 7A, 7B). Effective TRAP1-silencing and [A53T]α-Synuclein expression was confirmed by Western blot analysis (Figure 7C).

### Localization of [A53T]α-Synuclein to Mitochondria and Protein–Protein Interaction with TRAP1 in HEK293 Cell Culture

TRAP1 is defined as a mitochondrial molecular chaperone and has been shown to be protective against oxidative stress-induced cell death via multiple postulated mechanisms including cytochrome c release inhibition, interference with caspase-3 activation and attenuation of ROS production [39], [46], [48], [52]–[54]. We thus hypothesized that TRAP1 might directly antagonize α-Synuclein mitochondrial-related toxicity. Confirming that TRAP1 is indeed found in the mitochondria, co-localization studies in HEK293 cells revealed a strong overlap between TRAP1 staining with “Mitotracker Orange”-labeled mitochondria (Figure S7A). Therefore, it was interesting to see if α-Synuclein might also localize with mitochondria, as previously reported [33], [37], [38]. To determine this, we performed cell fractionation experiments to separate cytoplasmic and mitochondrial enriched fractions. Using Western blotting, these fractions and input control were compared for the content of endogenous, VDAC1 (mitochondrial outer membrane protein), β-Tubulin (cytosol) and α-Synuclein proteins. Whereas the input showed abundance of all tested proteins, the cytosolic fraction displayed expected cytosolic proteins β-Tubulin and α-Synuclein. In the mitochondria enriched fraction, no contaminating protein from β-Tubulin could be detected. Importantly, exogenous [A53T]α-Synuclein protein was found within the mitochondria enriched fraction (Figure S7B).

Given the strong rescue effect of TRAP1 on toxicity induced by [A53T]α-Synuclein in various systems (flies, primary rat neurons, and human cells), this implies at least a genetic interaction of these proteins. Whether there is a direct interaction of [A53T]α-Synuclein and TRAP1 awaits further analysis.

## Discussion

α-Synuclein plays an important role in PD pathogenesis. However, the mechanisms that actually lead to α-Synuclein-induced neurotoxicity remain unresolved. To gain insights into the disease mechanisms triggered by α-Synuclein, we performed a genome-wide modifier screen on [A53T]α-Synuclein-induced toxicity in flies. We used [A53T]α-Synuclein for our screen because its overexpression in flies results in a robust Parkinsonian phenotype [21], [55]–[57]. Toxicity induced by α-Synuclein or its mutant variants is rather low and eye-specific expression of A53T does not cause rough eye phenotypes (REPs). Such REPs induced by eye-specific expression of toxic proteins provide an excellent tool for screens and have successfully been used in the past to identify genetic interactions applying alterations in eye morphology due to photoreceptor degeneration as an endpoint. Given the low toxicity of [A53T]α-Synuclein, such screening approaches could not be conducted with regard to α-Synuclein-induced toxicity in flies. Our genetic screen fulfilled two important requirements: it utilized (i) an age-dependent model of [A53T]α-Synuclein toxicity, and (ii) an endpoint that is relevant to PD, this being the loss of DA. However, apart of being used as a neurotransmitter, DA in flies is also used for cuticle tanning. Thus, we cannot exclude the possibility that cuticle-derived DA might contribute to the overall DA in fly heads. Therefore, the measured decline is not only connected to DA loss in neurons. Nevertheless, secondary readouts like locomotion measurements or DA neuron counts indicate a strong correlation between decreased head DA content and proper function of DA neurons.

One of the candidates identified in our screen was the mitochondrial chaperone TRAP1. Consistent with our results, a genetic screen for alteration of α-Synuclein aggregation, conducted in *C. elegans*, identified *R151.7*, *a* homologue to *Drosophila* and human *TRAP1*, as a candidate worm gene. Knockdown of *R151.7* resulted in premature α-Synuclein aggregation [58]. Although aggregation was not assayed in our screen, this finding acts as an external confirmation that TRAP1 genetically interacts with α-Synuclein in different *in vivo* systems.

In multiple cell culture systems, TRAP1 has been shown to provide anti-apoptotic functions [48], [52], [53] as high levels of TRAP1 reduce the release of key factors involved in apoptosis, including Apoptosis Inducing Factor-1 and Cytochrome c, and additionally prevents Caspase-3 cleavage [39], [46], [48], [59]. The direct mechanisms by which TRAP1 might inhibit apoptosis were not examined in this study. However, given that overexpression of TRAP1 in both rat primary neurons and HEK293 cells was able to enhance cell survival after rotenone treatment, we hypothesize that anti-apoptotic mechanisms might in part be responsible for rescue of [A53T]α-Synuclein toxicity by TRAP1. This is in agreement with the observation that PD-associated neuronal death involves apoptotic cell death [60]–[63]. In addition, the effects of TRAP1 modulation on ATP synthesis and activities of the ETC support a mitochondrial function. For more than two decades, biochemical studies, the 1-methyl-4-phenyl-1,2,3,6-tetrahydropyridine (MPTP) and transgenic animal models have implicated mitochondrial dysfunction in the pathogenesis of PD [5], [64]–[71]. Genetic data, including mutations in PINK-1, Parkin, DJ-1, and HtrA2, have now specifically linked PD to both dysfunction and morphological change of the mitochondria [72]–[80]. However, the relationship of α-Synuclein pathology and mitochondrial dysfunction has been less clear. Our data are compatible with a localization of [A53T]α-Synuclein either in mitochondria or in mitochondrial membranes. Recent findings, though, have indicated that α-Synuclein may be localized to the outer mitochondrial membrane in pathological conditions and induce morphological changes of mitochondria by inhibiting mitochondrial fusion and enhancing mitochondrial fragmentation [51]. These morphological changes were rescued by overexpression of wild type PINK1, Parkin, and DJ-1 [51]. We show here that TRAP1 overexpression is also able to reverse [A53T]α-Synuclein-induced mitochondrial fragmentation.

Interestingly, TRAP1 has been identified as a substrate of the serine/threonine kinase PINK1. Phosphorylation of TRAP1 by PINK1 seems to be required for the protective effects mediated by PINK1. Combining these data with our findings leads to a potential pathogenic model, in which [A53T]α-Synuclein induces mitochondrial stress impairing, most likely, Complex I of the ECT by an as yet unidentified mechanism. Overexpression of TRAP1 counteracts this effect in flies, primary neurons and human neuronal as well as non-neuronal cells. TRAP1[D158N] is less effective in protecting from [A53T]α-Synuclein-induced detrimental effects. The finding suggests that a functional ATPase domain is required for TRAP1 function.

## Methods

### Fly Stocks

Flies were raised and maintained on standard cornmeal-yeast-molasses-agar food at 25°C unless otherwise noted. Non-RNAi stocks were obtained from the Bloomington *Drosophila* Stock Centre, UAS-RNAi stocks either from the Vienna *Drosophila* RNAi Center (VDRC) or from National Institute of Genetics (NIG-fly, Japan). Bloomington lines used were: Wild type flies (*Oregon R*; referred to as + in text), *w^1118^;; P{Ddc-GAL4.L}4.36* (BL7009; ddc: dopa decarboxylase, aminergic neuron specific driver, referred to in text as *ddc-GAL4*), w^*^; P{UAS-lacZ.B}Bg4-2-4b (BL1777; referred to in text as UAS-LacZ), *y^1^w^67c23^; P{SUPor-P}Trap1^KG06242^* (BL14032; referred to in text as TRAP1[KG]), w[*]; P{w[+mC] = longGMR-GAL4} (BL8605; referred to as GMR-GAL4 in text), *w[1118]; P{w[+mC] = UAS-HsapHSPA1L.W}53.1* (BL7455; expresses HSPA1L, the human homolog of HSP70 under GAL4 control; referred to as HSP70 in text), *w[1118]; P{w[+mC] = UAS-mitoGFP.AP}* (BL8443; expresses GFP with a mitochondrial import signal; referred to as mito-GFP in text) and w[*]; P{w[+mC] = UAS-Hsap\MJD.tr-Q78}c211.2 (BL8150; expresses a HA-tagged C-terminal fragment of the human Machado-Joseph Disease/Spinocerebellar Ataxia 3 protein with a 78 repeat polyglutamine tract; referred to in text as PolyQ). For stable eye-specific expression of PolyQ, GMR-GAL4 driver was recombined with PolyQ transgene (GMR>PolyQ in text). Fly lines suitable for GFP (*yw;; UAS-GFP*) or human [A53T]α-Synuclein (*yw;; UAS-[A53T]α-Synuclein* [41]) expression (referred to in text as GFP or A53T, respectively). Stable expression under control of the ddc driver (w[*];; ddc-GAL4>UAS-GFP; w[*];; ddc-GAL4>UAS-GFP) were generated by recombination (flies referred to in text as *ddc>GFP* or *ddc>A53T*).

Transgenic flies expressing human TRAP1 (hTRAP1) were generated by BestGene Inc. In brief, human TRAP1 cDNA was sub-cloned from pcDNA3.1+ vector into pUASattB using *KpnI* and *XbaI* restriction sites. hTRAP1 expression in these transgenic flies (*w;; UAS-hTRAP1/TM3*, *Sb*; referred to in text as hTRAP1) was verified by Western blotting.

### Genetic Deficiency Screen Breeding

The Bloomington Deficiency Kit was utilized for screening purposes (http://flystocks.bio.indiana.edu/Browse/df/dfkit_retired_July2009.htm). In general, a specific deficiency line was crossbred with *ddc>A53T* flies. Male offspring (*ddc>A53T* flies in combination with the respective deficiency) was selected and aged. At ages 1 and 4 weeks, a minimum of 9 flies was collected for later measurement of head DA using HPLC.

### Measurement of Fly Head Dopamine (DA) using HPLC

Liquid nitrogen flash frozen fly heads were homogenized (Precellys 24 homogenizer) in homogenization buffer (0.1 M perchloric acid/3% trichloric acid solution). 50 µl of supernatant from each sample were used for HPLC analysis (Dionex Ultimate 3000; running buffer: 57 mM citric acid, 43 mM sodium acetate, 0.1 mM EDTA, 1 mM octane sulfonic acid, 20% methanol). Samples were separated on a chromatographic column (Dionex Acclaim C18, 5 µm, 2.1×150 mm column, at 25°C), and DA was electrochemically detected on a graphite electrode (Dionex ED50 Electrochemical detector with following conditions: disposable carbon electrode at 0.8 V, flow rate 0.2 ml/min). DA (Sigma-Aldrich) standards of 0.1 µM, 0.25 µM and 0.4 µM were used for creation of a standard curve. Chromeleon 6.6 software was used for HPLC data analysis.

### Fly Longevity and Oxidative Stress Assays

Longevity assays were performed as previously described [81]. For oxidative stress assays a minimum of 20 male flies (2–3 days of age) was kept on filter papers soaked with paraquat (20 mM paraquat dichloride in 5% sucrose). Survival of flies was scored on a daily base. Fresh paraquat/sucrose solution was supplied daily.

### Negative Geotaxis Assay

Fly climbing was assessed in accordance with previously published protocols [21], . Flies were aged on normal yeast medium. At ages 1 and 4 weeks, climbing was assessed (20 flies per genotype). Flies were individually placed in a graduated cylinder, and allowed to climb for 15 s. Maximum height attained was recorded, and analysis was repeated 3 times per time point, with 3 trials at one minute intervals recorded at each time point.

### Immunohistochemistry

Fly brains were dissected in cold PBS, washed in a PBS/0.1% Triton X (PBT), fixed in 4% PFA (30 min, 4°C), and blocked in PBT containing 5% normal goat serum (overnight, 4°C). For TH staining, brains were incubated with primary anti-TH antibody (1∶100; rabbit polyclonal, AB152, Chemicon International/Millipore) for 2 days, 4°C, and subsequently with fluorescent secondary anti-rabbit antibody (1∶200; AlexaFluor-555 or Cy3; Invitrogen/Jackson Immunological Research) for 3.5 hours. Afterwards, brains were mounted in Vectashield (Vector Labs). The number of TH-positive neurons was determined on Z-stacked confocal sections (1 µm, Leica DM IRE2, Laser) [83]. At least 15 brains were analyzed per genotype.

### Protein Collection and Western Blotting

Fly heads were homogenized in RIPA buffer (50 mM Tris, pH 8.0, 0.15 M NaCl, 0.1% SDS, 1.0% NP-40, 0.5% Na-Deoxycholate, 2 mM EDTA, Complete Protease Inhibitors (Roche Applied Sciences), pH 7.4), centrifuged, and the supernatant was collected.

Cell culture protein samples were collected after washing cells in ice cold PBS, followed by lysis in RIPA buffer for 30 min on ice. Cell debris was removed by centrifugation, and supernatants were collected.

For Western blot analysis, protein samples were separated via SDS-PAGE gel and then transferred onto nitrocellulose membrane. Blocking in skim milk was followed by overnight primary antibody incubation. The primary antibodies used were as follows: mouse anti-α-Synuclein (1∶1000; Cell Signaling); mouse anti-*Drosophila* Syntaxin (1∶2000 Developmental Studies Hybridoma Bank (DSHB)), mouse anti-β-Tubulin (1∶500, DSHB); mouse anti-Δ Tubulin (1∶10,000; Sigma-Aldrich); mouse anti-TRAP1 (1∶1000; BD Biosystems); mouse anti-phospho-tyrosine (PY99) (1∶200; Santa Cruz Biotechnology); mouse anti-phospho-threonine (H2) (1∶200: Santa Cruz Biotechnology); mouse anti-phospho-serine (16B4) (1∶200; Santa Cruz Biotechnology); mouse anti-Cytochrome c (1∶500; Santa Cruz Biotechnology); rabbit anti-VDAC1 (0.3 µg/ml; Abcam); mouse anti-COX IV (2 µg/ml; Abcam); rabbit anti-GFP polyclonal (1∶1000; Santa Cruz Biotechnology).

Appropriate secondary anti-mouse or rabbit horseradish peroxidase-linked antibodies (1∶10,000) were obtained from GE Healthcare. Membranes were incubated with the secondary antibody for one hour, followed by signal detection using the Chemiglow substrate (Biozym).

### Total RNA Isolation, cDNA Preparation, and Real-Time PCR

Method for fly head RNA isolation was adapted from the following link: http://www.ou.edu/journals/dis/DIS84/Tec2%20Bertucci/Bertucci.htm. 20 fly heads per tube were used for RNA isolation. RNA samples were treated with DNase following manufacturer's instructions (Promega RQ1 RNase-Free DNase kit). Total RNA from cultured cells was prepared from cells using Qiagen RNeasy Mini kit (Qiagen). RNA was used for cDNA production via reverse transcription using the iScript cDNA Synthesis Kit (BioRad). Real-time PCR measurements were performed using the SYBR Green (Thermo Fisher Scientific) reagent following manufacturer's instructions for preparation of PCR samples. Gene of interest signal was compared to that of control gene expression (β-Actin5c for fly samples and 18S for human samples) using the 2^−ΔΔCt^ method [84]. No-RT controls were performed to exclude for genomic DNA sample contamination. PCR reactions were followed by generation of a dissociation curve to check for side product generation.

Amplification conditions for fly samples were as follows: 5 min at 95°C, 40 cycles of: 30 s at 95°C, 30 s at 58°C, 60 s at 72°C, followed by 10 min at 72°C. Gene of interest was normalized to control β-Actin5c signal. The primers used are listed in Table S5.

Amplification conditions for cell culture samples were as follows: 5 min at 95°C, 40 cycles of: 30 s at 94°C, 30 s at 60°C, 60 s at 72°C, followed by 10 min at 72°C. Gene of interest was normalized to control 18S signal. For primers, see Table S5.

### Cell Culture

#### Cloning and *in vitro* mutagenesis

Full length human α-Synuclein cDNA (423 bp), carrying the [A53T] mutation (cDNA a gift from Dr. Felipe Opazo, European Neuroscience Institute, Göttingen, Germany) was subcloned into the pcDNA3.1+ expression vector (Invitrogen) using *HindIII* and *EcoRV* restriction sites. Full length human TRAP1 cDNA (2115 bp) was amplified from human HEK293 cell cDNA samples. *BglII* and *XhoI* restriction sites were introduced using primers listed in Table S5.

TRAP1 cDNA was then cloned into the pcDNA3.1+ vector using the *BglII*, *XhoI* sites.

*In vitro* mutagenesis of human TRAP1 in pcDNA3.1+ was carried out using the QuickChange Site-Directed Mutagenesis kit (Stratagene) following manufacturer's instructions. Sites for mutagenesis were based on conserved sites found in the ATPase domain (see Figure S6 for sequence homology). PCR cycling parameters were used as suggested by Stratagene, with a specific extension time of 8 min and 16 cycles for all reactions. Primers used for generating the mutants are listed in Table S5.

#### Lentivirus preparation

Full length human TRAP1 or [A53T]α-Synuclein cDNA was subcloned into a third generation lentiviral vector pRRLsin.cPPT.PGK/GFP.WPRE (Tronolab), excluding the GFP cassette. The GFP-expressing virus served as a control [85].

#### Cells, plasmid transfection, and viral infection

Human HEK293 cells were grown in Dulbecco's modified Eagle's medium (DMEM), supplemented with 10% fetal calf serum, 100 units/ml penicillin, and 100 mg/ml streptomycin. Transfection of plasmids and small interfering RNA (siRNA) into HEK293 cells was completed using Metafectene (Biontex) following manufacturer's instructions. siRNAs used for gene knockdown experiments were obtained from Qiagen: MAPK1 control siRNA (Qiagen 1027277); 2 different TRAP1 siRNAs (Flexitube siRNA SI03066364 and siRNA SI00115150); a scrambled siRNA for control (Allstars Negative Control, 1027280). Final concentration of siRNA used was 10 nM. Cells were seeded on poly-L lysine (PLL) coated plates (35,000 cells/cm^2^) and then transfected 48 hours before experimentation.

Primary cortical rat neurons were prepared from E18 rat embryos, following previously published procedures [65]. Neurons were seeded on poly-ornithin-coated 24-well plates at a density of 125,000 cells/cm^2^. Cells were maintained in Neurobasal medium (Gibco/Invitrogen), supplemented with 5 µg/ml transferrin, 1% PSN, 0.5 mM L-Glutamine, 2% B27 supplement. Primary neurons were infected equimolarly with lentiviruses one day after isolation and then cultured for 6 days before experimental use.

SH-SY5Y cells (DSMZ number ACC 209) were cultured in DMEM F-12 with glutamine (Lonza) supplemented with 15% (v/v) fetal calf serum, non-essential amino acids (Invitrogen) and penicillin/streptomycin. Transfections were performed using Lipofectamine Plus (Invitrogen) according to the manufacturer's instructions. The following plasmids were described earlier: [A53T]α-Synuclein and mito-DsRed [51]. For downregulation of TRAP1, SH-SY5Y cells were reversely transfected with the indicated siRNA and co-transfected with mito-DsRed and empty vector or [A53T]α-Synuclein using Lipofectamin RNAiMax (Invitrogen) according to the manufacturers instruction. 24 hours after transfection, fresh medium was added. Cells were analyzed 48 h after transfection.

#### Fluorescent staining of mitochondria

SH-SY5Y cells were plated on 15 mm glass coverslips and co-transfected with mito-DsRed and the indicated DNA constructs. At 24 h after transfection, cells were washed twice with ice-cold PBS, fixed with 3.7% paraformaldehyde for 10 minutes at room temperature and washed twice with PBS before mounting the coverslips. Transfected cells were identified by co-expression of mito-DsRed. Cells were categorized in two classes according to their mitochondrial morphology [75]. Cells displaying an intact network of tubular mitochondria were classified as tubular. When this network was disrupted and mitochondria appeared predominantly spherical or rod-like, they were classified as fragmented. The mitochondrial morphology of at least 300 cells per coverslip was determined in a blinded manner using a Leica DMRB microscope. Quantifications were based on triplicates of at least three independent experiments. Confocal images of representative cells were obtained using a Zeiss LSM 510 microscope. α-Synuclein was detected using a monoclonal anti-rat antibody described previously [51]. β-Actin was detected using a monoclonal antibody from Sigma.

### Cell Culture Oxidative Stress Testing and Measurement of Cell Viability

Cells were incubated for 16 hours in the presence of either hydrogen peroxide (100 µM) or rotenone in DMSO (HEK293: 200 µM, rat cortical: 1 µM rotenone). Rotenone control cells were treated with equivalent amount of DMSO alone. After overnight oxidative stress treatment, cells were fixed in 4% PFA for 10 min, before permeabilization in PBT for 10 min. Cells were incubated with Hoechst nuclear stain for 30 min before imaging on a fluorescent microscope (Leica DMI6000B). Using a macro within the Leica Qwin V3 quantification software, cell number remaining in each well was assessed by counting total fluorescent nuclei (6 images per well at 10×, with minimum 6 wells per genotype in a 24-well culture dish).

### ATP Synthesis Assay

Method was adapted from [86]. Cells were trypsinized and washed three times in cold PBS. Cells were then resuspended in incubation medium (2×10^5^ cells/ml, 25 mM Tris, 125 mM KCl, 2 mM K+EDTA, 10 mM KH_2_PO_4_, pH 7.4) and permeabilized with digitonin (40 µg/ml). To measure ATP produced via complexes I, III, IV, 2×10^4^ cells (minimum of 8 replicates per experiment) were resuspended in a complex incubation medium supplemented with 1 mg/ml BSA, 2 mM ADP, 10 mM glutamate, 2 mM malate. After incubation for 20 min at 37°C, the reaction was stopped by addition of 6% perchloric acid, and samples were neutralized with 3 M K_2_CO_3_. ATP in each sample was measured in a plate reader using the CellTiter-Glo Luminescent reagent (Promega) following manufacturer-provided instructions. Total ATP levels were obtained using an ATP standard curve (ATP from Sigma), with final calculation expressing ATP as pmoles ATP per minute per 1 million cells, and plotted as percentage of control values.

### Measurement of Steady State Cellular ATP

2×10^4^ HEK293 cells/well were seeded (12 replicates per experiment) in an opaque white 96-well plate. Total ATP levels were measured as stated above.

### Assessment of Mitochondrial Membrane Potential

Following manufacturer-provided instructions, cells (6 replicates per experiment) in a 96-well plate (black sided, clear bottom) were incubated with the mitochondrial probe JC-1 (3 µg/ml; Invitrogen) in full medium for 30 min at 37°C. After washing in PBS, JC-1 mitochondrial aggregates were measured in a plate reader (excitation: 530 nm, emission: 590 nm). As a control, JC-1 fluorescence was measured in the presence of the mitochondrial membrane potential inhibitor CCCP (Carbonyl cyanide *m*-chlorophenylhydrazone, 50 µM). Protein content per well was quantified using the Bradford assay. Fluorescence in relation to total protein was displayed as percentage of control values.

### Immunocytochemistry

Cells were plated on PLL-coated glass slips. 48 hours after transfection, cells were fixed in 4% PFA for 10 min, permeabilized in PBT for 10 min, followed by blocking in 1% BSA and overnight incubation with the primary antibody at 4°C. Antibodies used: monoclonal rat anti-α-Synuclein (1∶500, Alexis Biochemicals/Enzo Life Sciences); monoclonal mouse anti-NeuN (1∶500, Chemicon); monoclonal mouse anti-TRAP1 (1∶300, Alexis Biochemicals). For visualization of mitochondria cells pretreated for 4 hours with 1 µM rotenone, the cells were incubated with Mitotracker Orange CMTMRos (300 nM, following manufacturer's instructions, Invitrogen) for 30 min at 37°C prior to fixation. Cells were incubated with respective secondary antibodies for one hour (all secondary antibodies 1∶1000, anti-mouse or rat AlexaFluor-488, 543, 633, Invitrogen), and mounted using Mowiol (Calbiochem), with or without the anti-bleaching agent DABCO or nuclear stain Hoechst (Sigma-Aldrich).

### Mitochondrial Isolation

Mitochondria were isolated from HEK293 cells transfected with [A53T]α-Synuclein using the following protocol: Cells were suspended in MB buffer (70 mM sucrose, 10 mM HEPES, 1 mM EDTA, 210 nM Mannitol (pH 7.5) and protease inhibitors), homogenized with an injection needle (27G ¾″ 19 mm, 5–6 strokes) and centrifuged at 750×g for 7 min. After centrifugation the pellet was resuspended in MB buffer, homogenized using the same injection needle and centrifuged. This procedure was repeated four times. The resulting supernatants were pooled and centrifuged at 10000×g for 30 min. This mitochondria-containing pellet was resuspended in MB buffer and further centrifugated at 1500×g for 20 min. The purity of the resulting mitochondrial pellet was examined by Western blotting using specific antibodies directed against β-Tubulin, VDAC and α-Synuclein.

### Statistics

Data was analyzed using GraphPad Prism 4.0 (GraphPad Software Inc.), using 1-way ANOVA followed by Newman-Keuls post testing. Use of a 2-way ANOVA was noted in the text. Survival data were analyzed with the Kaplan-Meier analysis method and the Log Rank Test for curve statistical comparison analysis. Statistical significance referred to as: *p<0.05; **p<0.01; ***p<0.001. All data is presented as mean ± SEM.

## Supporting Information

Figure S1 Validation, specificity and sensitivity of HPLC to measure fly head DA. (A) Number of fly heads for single measurement varied from 3–30 and absolute DA amounts measured by HPLC were analyzed via linear regression; r^2^ = 0.997 (n = 3). (B) Fly heads collected at indicated time of day were analyzed for DA using HPLC. Significant difference (ANOVA followed by Newman-Keuls Multiple Comparison Test) between time points noted: *p<0.05 vs. 10∶30 and 16∶30 (n = 3). (C) Wild type flies (one day post eclosion) were daily treated with tyrosine hydroxylase inhibitor α-methyltyrosine (α-MT). Fly heads were collected at 2-day intervals for measurement of DA using HPLC: Significant differences (t-test): *p<0.05 for Control (untreated) vs. 5 mM α-MT (n = 3).
(JPG)

Figure S2 Determination of TRAP1 transcript abundance. Flies heterozygous for P-element insertion TRAP1[KG] displayed a significant reduction in *trap1* transcript levels normalized to *actin5C* independent of *[A53T]α-Synuclein* expression (t-test, compared to respective control; **p<0.01; ***p<0.001).
(JPG)

Figure S3 Immunocytochemistry of [A53T]α-Synuclein-expressing HEK293 cells with overexpression or downregulation of TRAP1. (A) HEK293 cells transfected with [A53T]α-Synuclein were stained for TRAP1 (red), α-Synuclein (green) and DNA (blue). Merged picture is shown (right column). Upper panel: [A53T]α-Synuclein expressing cells co-transfected with empty vector. Middle panel: [A53T]α-Synuclein expressing cells with siTRAP1. Lower panel: Cells with [A53T]α-Synuclein and TRAP1 overexpression (scale bar = 24 µm). (B) Transfection with siTRAP1 reduced endogenous TRAP1 transcripts (in relation to β-Actin). (C) Both siTRAP1-1 and siTRAP1-2 resulted in significant knockdown of TRAP1 expression (ANOVA followed by Newman-Keuls Multiple Comparison Test; n = 3; ***p<0.001; ns = not significant).
(JPG)

Figure S4 Assessment of mitochondrial proteins. Changes in mitochondrial function by (A) alterations of TRAP1 levels or (B) expression of mutant TRAP1[D158N] are not caused by a decrease in overall mitochondrial load. Western blot analysis of HEK293 transfected with indicated constructs were assayed for abundance of the mitochondrial proteins VDAC1 and COX4. β-Actin served as loading control.
(JPG)

Figure S5 Stable silencing of TRAP1 causes a reduction in membrane potential of [A53T]α-Synuclein-expressing cells. We stably silenced TRAP1 in HEK293 cells using Lentiviruses-expressing short hairpin RNA (shRNA). (A) Silencing of TRAP1 was verified by Western blot. In contrast to HEK cells with stable expression of a scrambled shRNA construct, TRAP1-silenced cells displayed a strong reduction of TRAP1 protein load. Quantification of Western blots (n≥3) normalized with either VDAC or Tubulin revealed a strong reduction of TRAP1 in cells expressing shTRAP1 (95.43+/−1.25%). The presence of similar amounts of VDAC in relation to Tubulin between scrambled shRNA-expressing and TRAP1-silenced cells indicates that mitochondrial load is not effected by shTRAP1. (B) Membrane potential was measured using the dye TMRM. Cells with stable expression of either of scrambled shRNA or TRAP1 shRNA were co-transfected with pEPFP-N1, in combination with pCDNA3.1 or pCDNA3.1-[A53T]α-Synuclein. 2 days after transfection cells were treated with 200 nM TMRM for 30 minutes at 37°C. Fluorescence was measured at 573 nm (TMRM) and 509 nm (EGFP) and plotted as relative intensity (TMRM/GFP). Cells expressing scrambled shRNA displayed non-significant (ns) changes in membrane potential with or without [A53T]α-Synuclein-expression. In contrast, expression of [A53T]α-Synuclein caused a significant reduction in membrane potential of TRAP1-silenced cells (***p<0.001). Statistic: 2-way ANOVA followed by Bonferroni post-hoc tests.
(JPG)

Figure S6 Protein sequence data for TRAP1 used for mutant generation. (A) Protein sequence alignment showing conserved aspartic acid in HSP90 ATPase domains. The indicated conserved amino acid is reported to be critical for ATPase function in yeast Hsp82. Moreover, this aspartic acid is conserved in ATPase domains of human TRAP1 (position 158). (B) Multiple sequence comparison of TRAP1 proteins from different species showed a high degree of conservation of this aspartic acid in the ATPase domain. Alignments were performed using ClustalW2 (http://www.ebi.ac.uk/Tools/msa/clustalw2/).
(JPG)

Figure S7 Localization of TRAP1 and [A53T]α-Synuclein to the mitochondria. (A) Mitochondrial localization of TRAP1. Confocal section of HEK293 cells stained with the mitochondrial marker “Mitotracker Orange” (red), hTRAP1-specific antibody (green) and Hoechst nuclear stain (blue). A high degree of co-localization of red and green fluorescent signals is apparent in overlay. Scale bar indicates 27 µm. (B) Cell fractionation assay indicates localization of [A53T]α-Synuclein in mitochondria-enriched fraction. Samples derived after fractionation were used for Western blot analysis. Blots were probed with specific antibodies detecting α-Synuclein, VDAC1 and ß-Tubulin. Fractions analyzed (input, cytoplasmic and mitochondrial-enriched fraction) are indicated.
(JPG)

Figure S8 Detailed statistical analysis of the data shown in Figure 4. Summary of statistical analysis of bar graphs in Figure 4A–4C (ANOVA followed by Newman-Keuls Multiple Comparison Test). *p<0.05; **p<0.01; ***p<0.001; ns = not significant.
(JPG)

Table S1 List of deficiencies identified to cause haploinsufficiency with *ddc>A53T.*
(PDF)

Table S2 List of deficiencies identified to cause semi-lethality.
(PDF)

Table S3 Detailed summary of analyzed genes located within deficiencies causing a haploinsufficiency in combination with *ddc>A53T.*
(PDF)

Table S4 Deficiencies causing a decline in DA.
(PDF)

Table S5 Primers.
(PDF)

Text S1 Brief summary of screen results.
(DOC)
