# Supplementary figures and images for: The Mitochondrial Chaperone Protein TRAP1 Mitigates α-Synuclein Toxicity
Source: PLoS Genet. 2012 Feb 2;8(2):e1002488. doi: 10.1371/journal.pgen.1002488 (PMC3271059; doi:10.1371/journal.pgen.1002488)

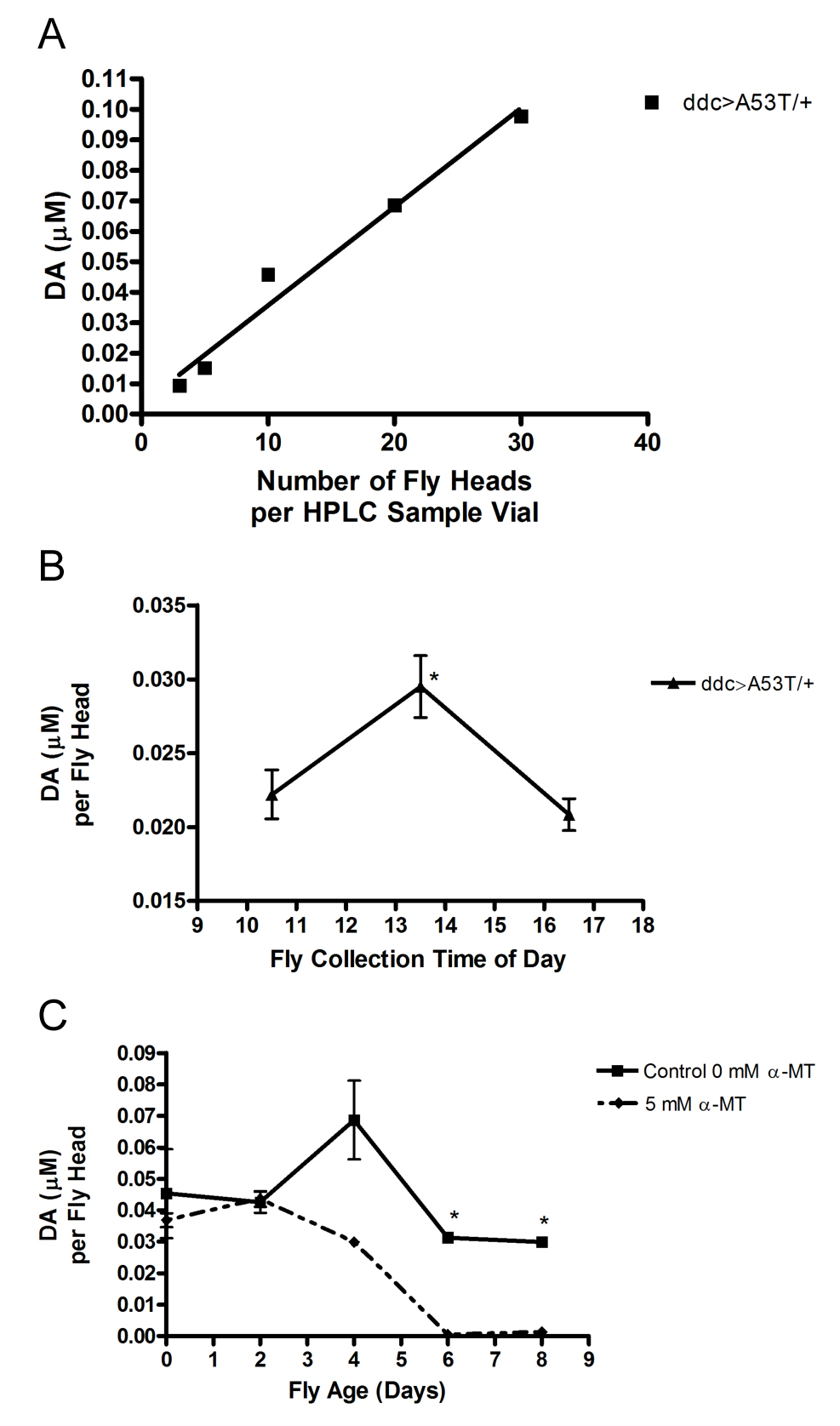

Supplement: Figure S1 — Validation, specificity and sensitivity of HPLC to measure fly head DA. (A) Number of fly heads for single measurement varied from 3–30 and absolute DA amounts measured by HPLC were analyzed via linear regression; r2 = 0.997 (n = 3). (B) Fly heads collected at indicated time of day were analyzed for DA using HPLC. Significant difference (ANOVA followed by Newman-Keuls Multiple Comparison Test) between time points noted: *p<0.05 vs. 10∶30 and 16∶30 (n = 3). (C) Wild type flies (one day post eclosion) were daily treated with tyrosine hydroxylase inhibitor α-methyltyrosine (α-MT). Fly heads were collected at 2-day intervals for measurement of DA using HPLC: Significant differences (t-test): *p<0.05 for Control (untreated) vs. 5 mM α-MT (n = 3). (JPG) [file pgen.1002488.s001.jpg]

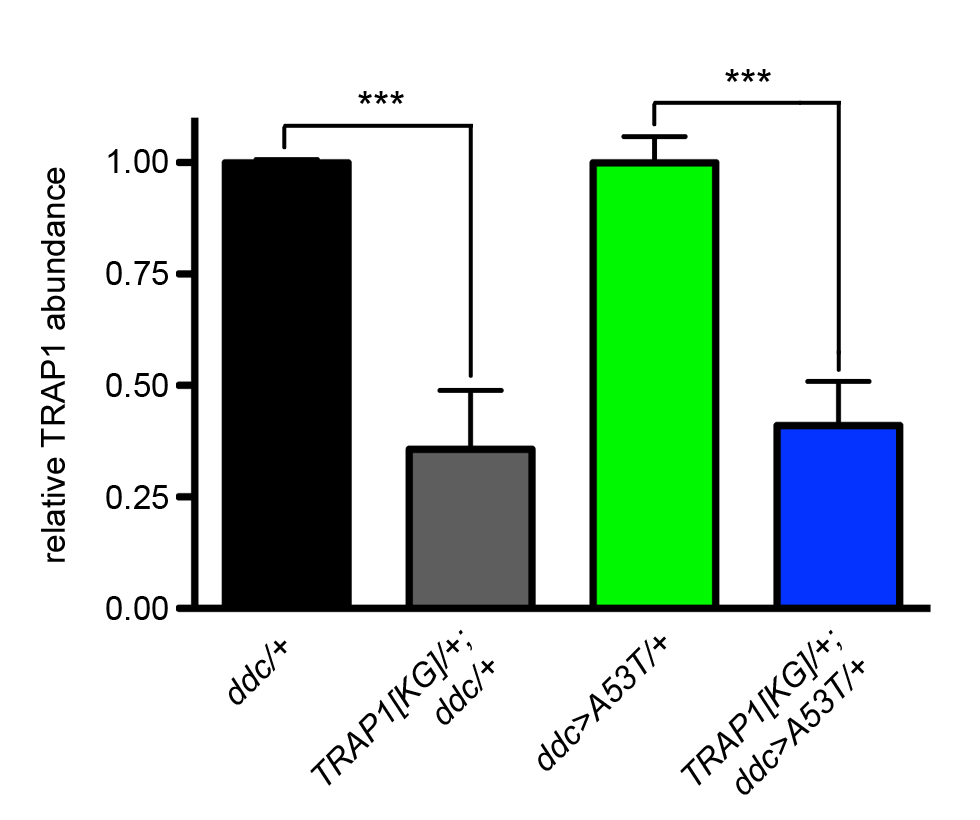

Supplement: Figure S2 — Determination of TRAP1 transcript abundance. Flies heterozygous for P-element insertion TRAP1[KG] displayed a significant reduction in trap1 transcript levels normalized to actin5C independent of [A53T]α-Synuclein expression (t-test, compared to respective control; **p<0.01; ***p<0.001). (JPG) [file pgen.1002488.s002.jpg]

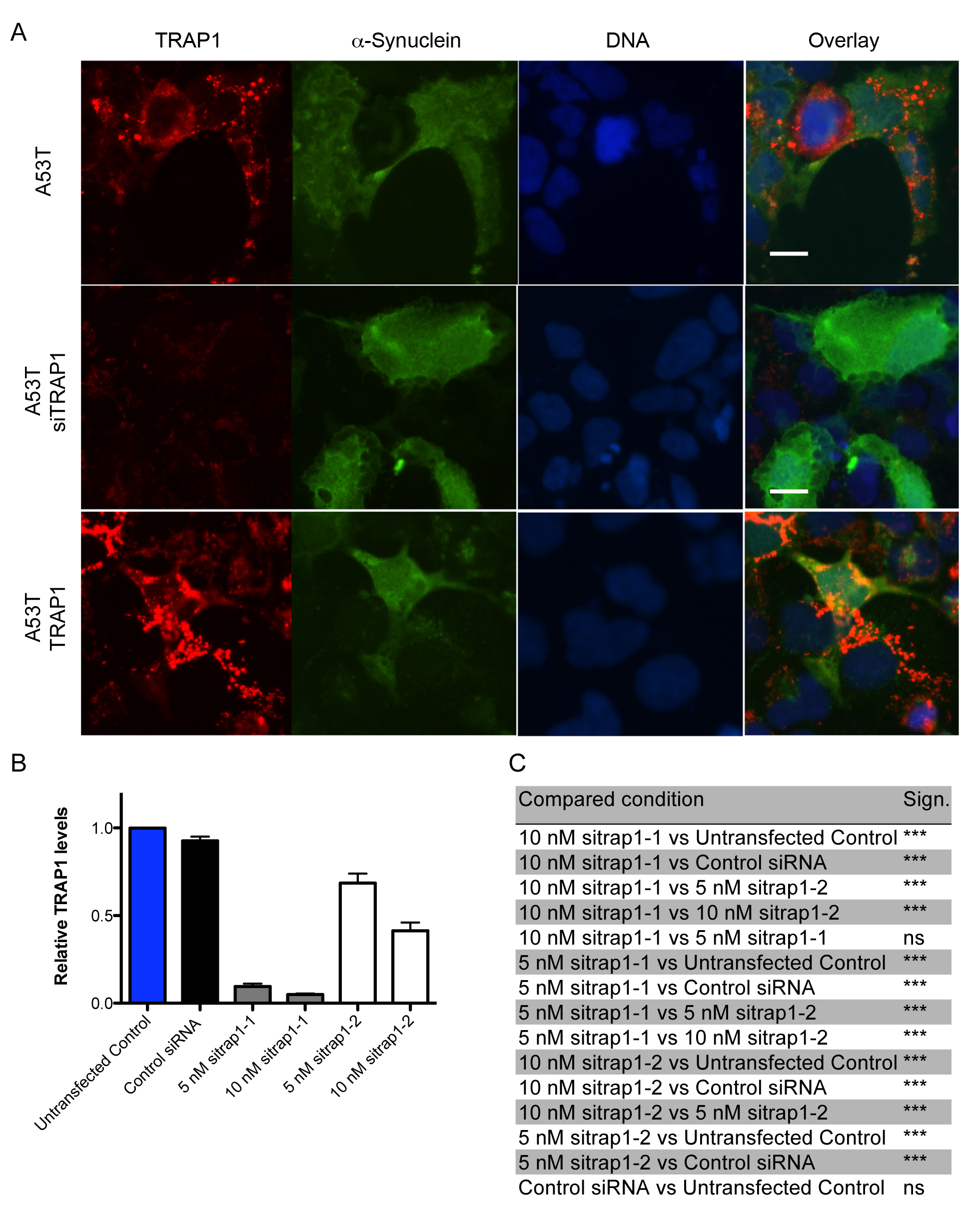

Supplement: Figure S3 — Immunocytochemistry of [A53T]α-Synuclein-expressing HEK293 cells with overexpression or downregulation of TRAP1. (A) HEK293 cells transfected with [A53T]α-Synuclein were stained for TRAP1 (red), α-Synuclein (green) and DNA (blue). Merged picture is shown (right column). Upper panel: [A53T]α-Synuclein expressing cells co-transfected with empty vector. Middle panel: [A53T]α-Synuclein expressing cells with siTRAP1. Lower panel: Cells with [A53T]α-Synuclein and TRAP1 overexpression (scale bar = 24 µm). (B) Transfection with siTRAP1 reduced endogenous TRAP1 transcripts (in relation to β-Actin). (C) Both siTRAP1-1 and siTRAP1-2 resulted in significant knockdown of TRAP1 expression (ANOVA followed by Newman-Keuls Multiple Comparison Test; n = 3; ***p<0.001; ns = not significant). (JPG) [file pgen.1002488.s003.jpg]

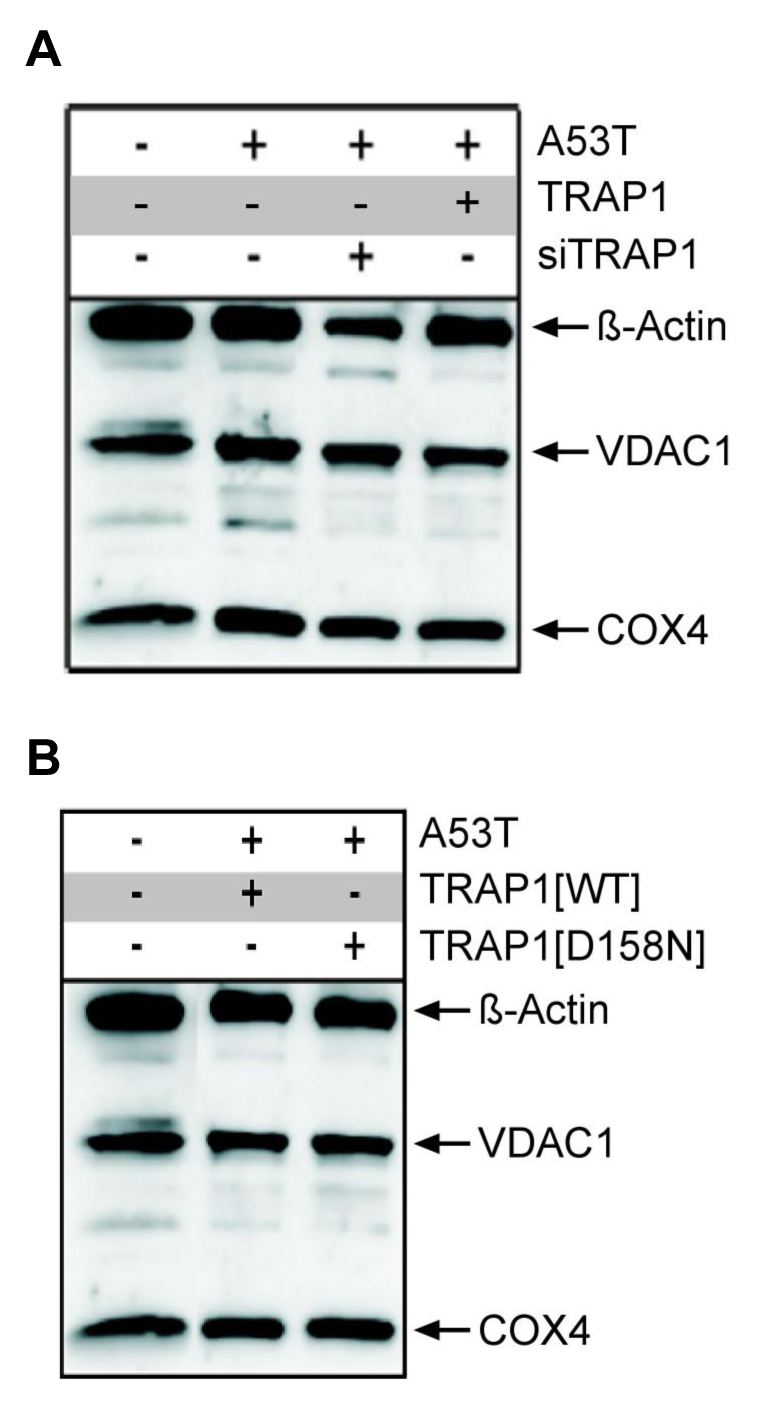

Supplement: Figure S4 — Assessment of mitochondrial proteins. Changes in mitochondrial function by (A) alterations of TRAP1 levels or (B) expression of mutant TRAP1[D158N] are not caused by a decrease in overall mitochondrial load. Western blot analysis of HEK293 transfected with indicated constructs were assayed for abundance of the mitochondrial proteins VDAC1 and COX4. β-Actin served as loading control. (JPG) [file pgen.1002488.s004.jpg]

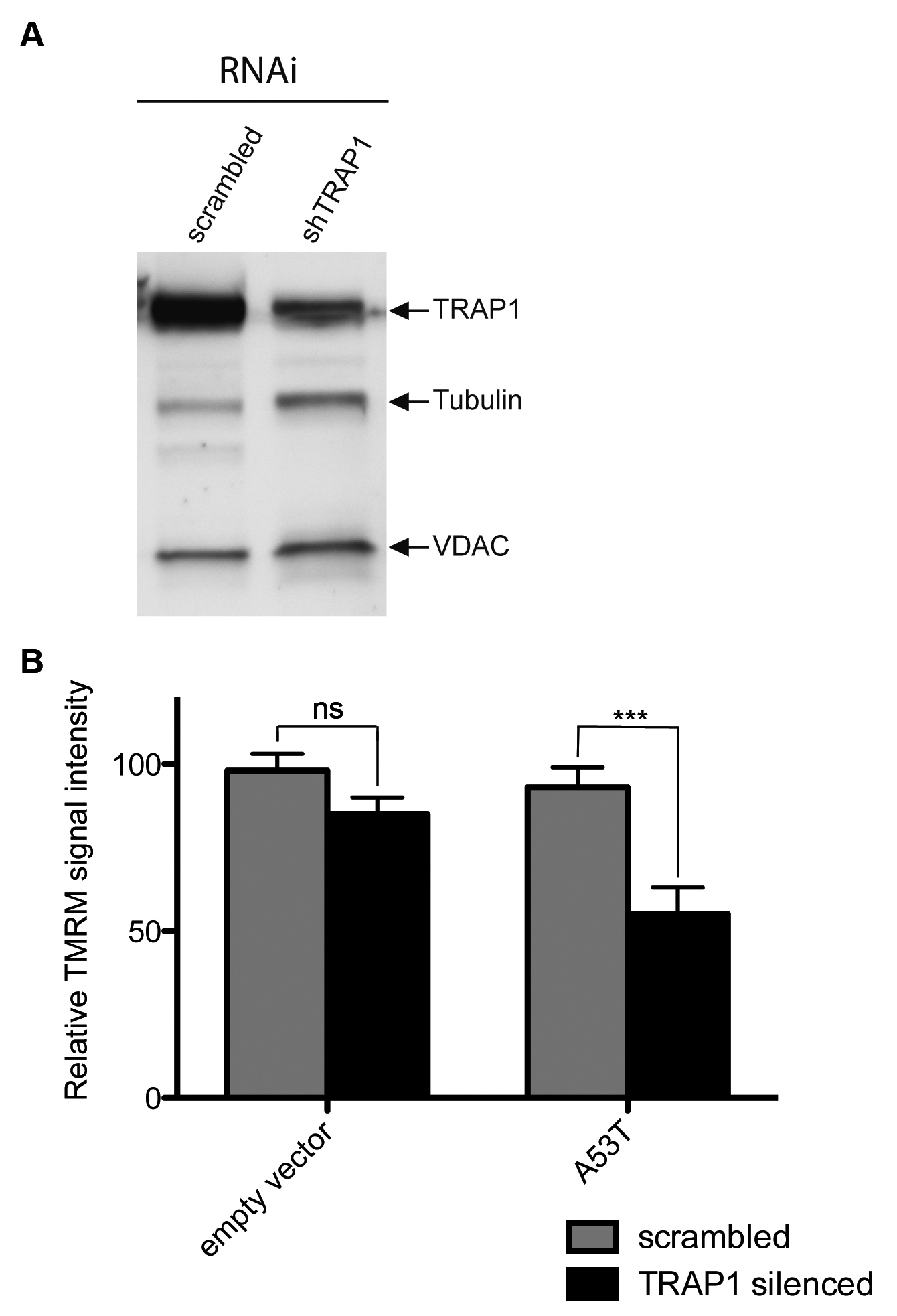

Supplement: Figure S5 — Stable silencing of TRAP1 causes a reduction in membrane potential of [A53T]α-Synuclein-expressing cells. We stably silenced TRAP1 in HEK293 cells using Lentiviruses-expressing short hairpin RNA (shRNA). (A) Silencing of TRAP1 was verified by Western blot. In contrast to HEK cells with stable expression of a scrambled shRNA construct, TRAP1-silenced cells displayed a strong reduction of TRAP1 protein load. Quantification of Western blots (n≥3) normalized with either VDAC or Tubulin revealed a strong reduction of TRAP1 in cells expressing shTRAP1 (95.43+/−1.25%). The presence of similar amounts of VDAC in relation to Tubulin between scrambled shRNA-expressing and TRAP1-silenced cells indicates that mitochondrial load is not effected by shTRAP1. (B) Membrane potential was measured using the dye TMRM. Cells with stable expression of either of scrambled shRNA or TRAP1 shRNA were co-transfected with pEPFP-N1, in combination with pCDNA3.1 or pCDNA3.1-[A53T]α-Synuclein. 2 days after transfection cells were treated with 200 nM TMRM for 30 minutes at 37°C. Fluorescence was measured at 573 nm (TMRM) and 509 nm (EGFP) and plotted as relative intensity (TMRM/GFP). Cells expressing scrambled shRNA displayed non-significant (ns) changes in membrane potential with or without [A53T]α-Synuclein-expression. In contrast, expression of [A53T]α-Synuclein caused a significant reduction in membrane potential of TRAP1-silenced cells (***p<0.001). Statistic: 2-way ANOVA followed by Bonferroni post-hoc tests. (JPG) [file pgen.1002488.s005.jpg]

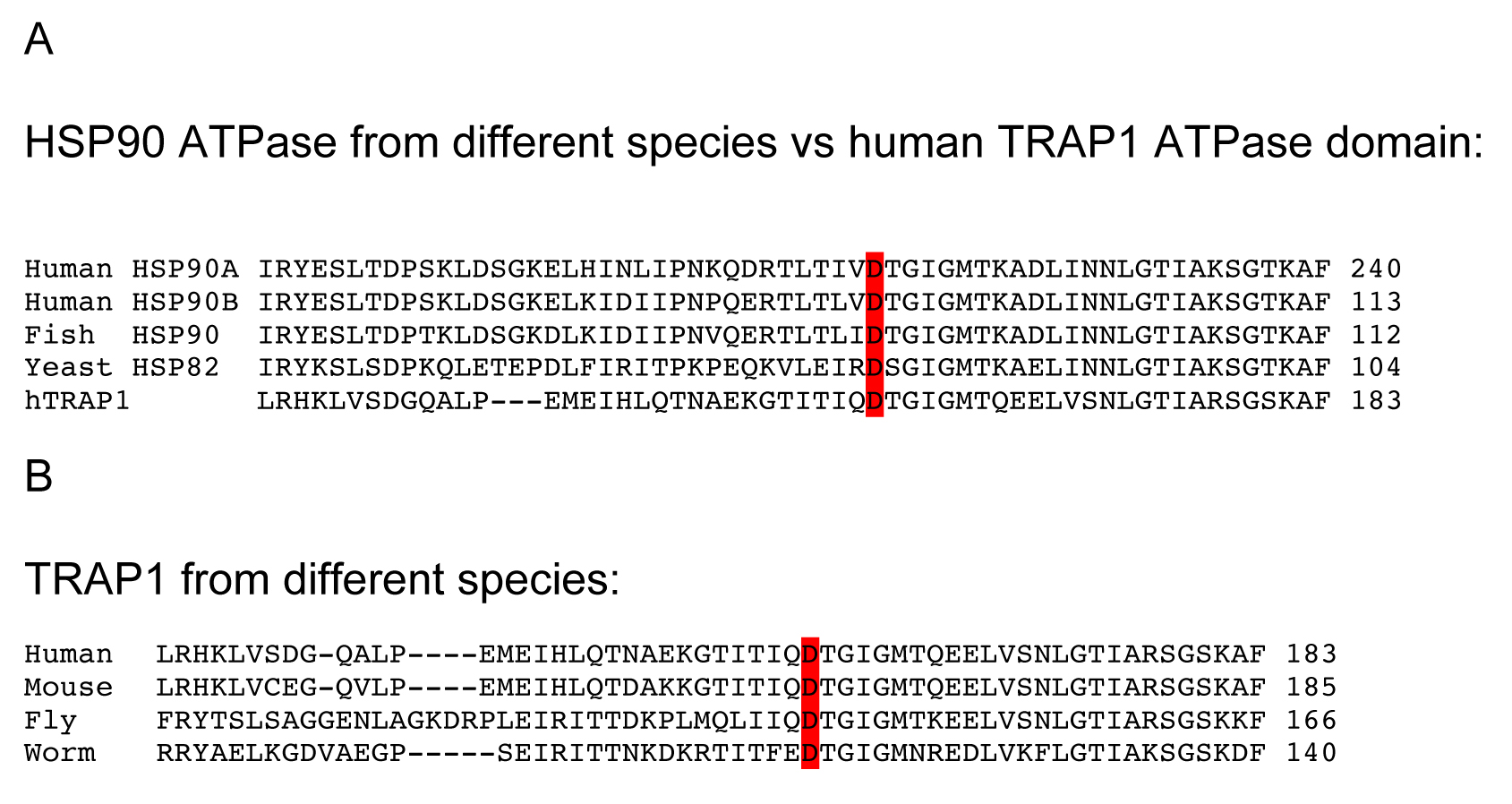

Supplement: Figure S6 — Protein sequence data for TRAP1 used for mutant generation. (A) Protein sequence alignment showing conserved aspartic acid in HSP90 ATPase domains. The indicated conserved amino acid is reported to be critical for ATPase function in yeast Hsp82. Moreover, this aspartic acid is conserved in ATPase domains of human TRAP1 (position 158). (B) Multiple sequence comparison of TRAP1 proteins from different species showed a high degree of conservation of this aspartic acid in the ATPase domain. Alignments were performed using ClustalW2 (http://www.ebi.ac.uk/Tools/msa/clustalw2/). (JPG) [file pgen.1002488.s006.jpg]

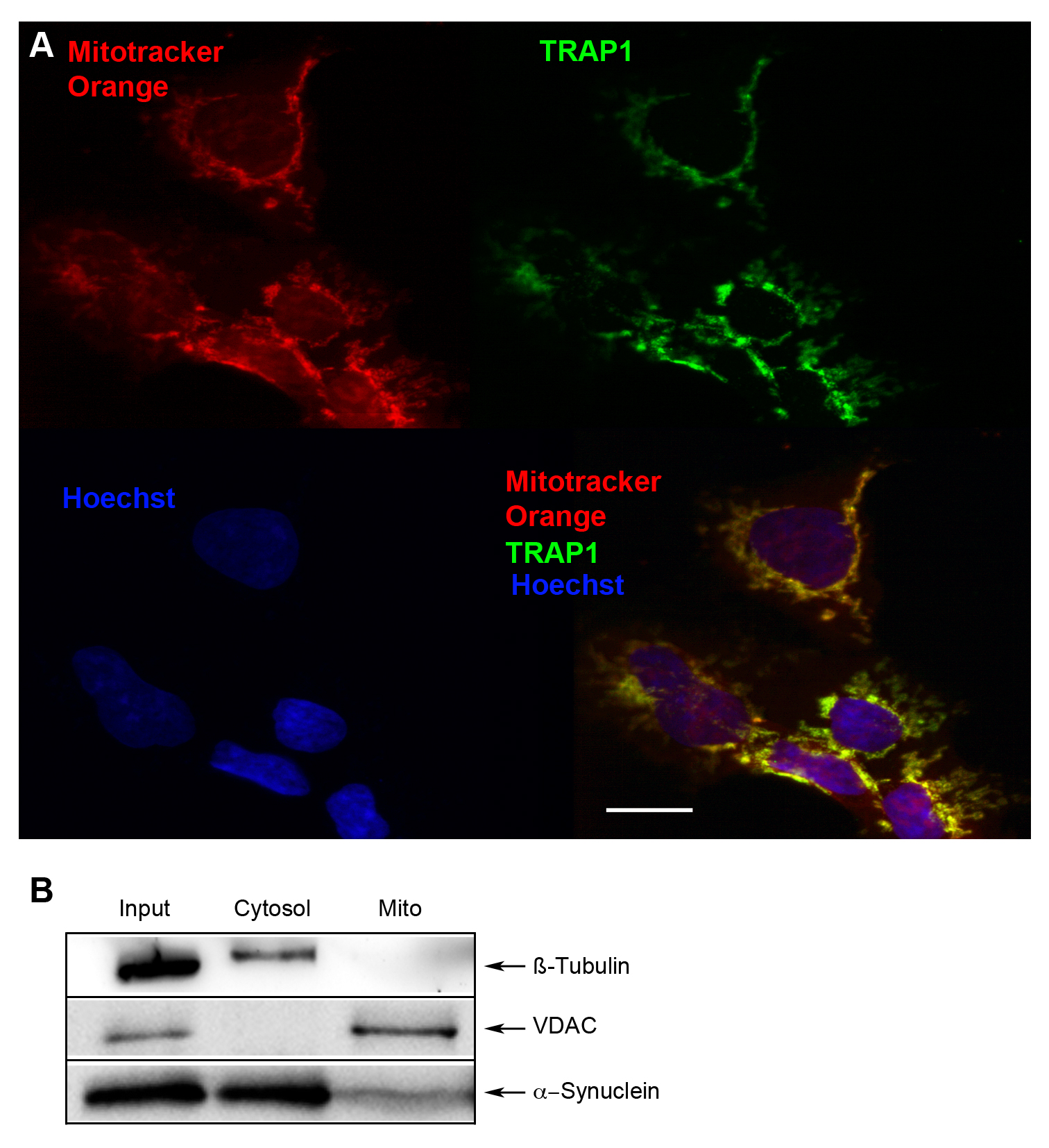

Supplement: Figure S7 — Localization of TRAP1 and [A53T]α-Synuclein to the mitochondria. (A) Mitochondrial localization of TRAP1. Confocal section of HEK293 cells stained with the mitochondrial marker “Mitotracker Orange” (red), hTRAP1-specific antibody (green) and Hoechst nuclear stain (blue). A high degree of co-localization of red and green fluorescent signals is apparent in overlay. Scale bar indicates 27 µm. (B) Cell fractionation assay indicates localization of [A53T]α-Synuclein in mitochondria-enriched fraction. Samples derived after fractionation were used for Western blot analysis. Blots were probed with specific antibodies detecting α-Synuclein, VDAC1 and ß-Tubulin. Fractions analyzed (input, cytoplasmic and mitochondrial-enriched fraction) are indicated. (JPG) [file pgen.1002488.s007.jpg]

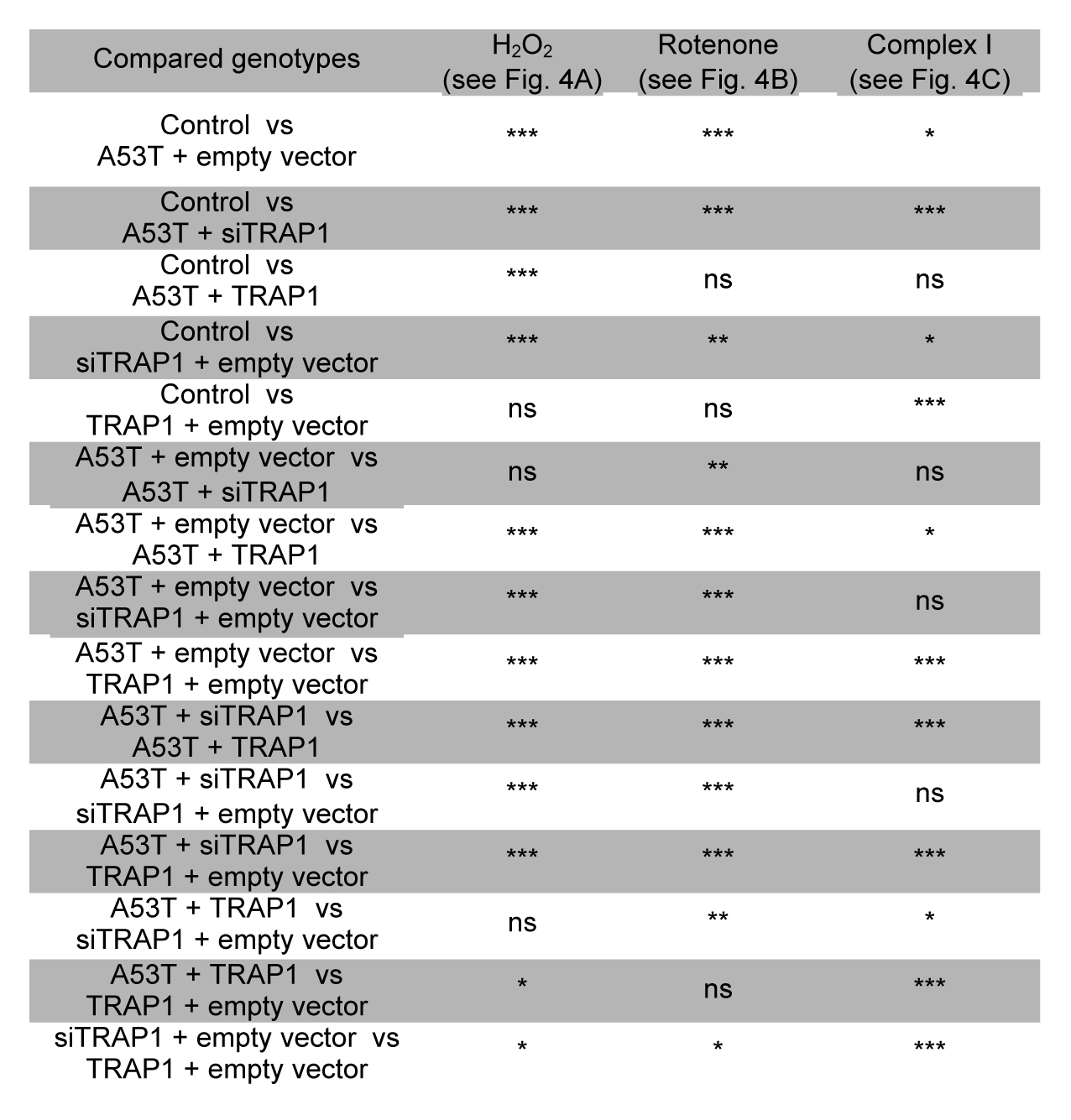

Supplement: Figure S8 — Detailed statistical analysis of the data shown in Figure 4. Summary of statistical analysis of bar graphs in Figure 4A–4C (ANOVA followed by Newman-Keuls Multiple Comparison Test). *p<0.05; **p<0.01; ***p<0.001; ns = not significant. (JPG) [file pgen.1002488.s008.jpg]
