# Supplementary material for: The Mitochondrial Chaperone Protein TRAP1 Mitigates α-Synuclein Toxicity
Source: PLoS Genet. 2012 Feb 2;8(2):e1002488. doi: 10.1371/journal.pgen.1002488 (PMC3271059; doi:10.1371/journal.pgen.1002488)
Supplement: Table S1 — List of deficiencies identified to cause haploinsufficiency with ddc>A53T. (PDF) [file pgen.1002488.s009.pdf]

Table S1. List of deficiencies identified to cause haploinsufficiency with *ddc>A53T*

Deficiency	Candidate region	Genes in deleted region	Genes analyzed	Confirmed synthetic haploinsufficiency
Df(1)dx81	5C3-6C12	122	62 (51%)	*kdn*
Df(1)BK10	15F2-16C10	51	36 (69%)	-
*Df(2L)Exel6011**	25C7-25D5	31	15 (48%)	-
Df(3L)h-i22	66D10-66D12	13	12 (92%)	-
*Df(3R)e-R1**	93B7-93D3	51	39 (76%)	-

* These deficiencies cause synthetic haploinsufficiencies with various other disease models (but not with driver only controls). Consequently, the focus of our analysis was on the remaining candidates.
