# Supplementary material for: The Mitochondrial Chaperone Protein TRAP1 Mitigates α-Synuclein Toxicity
Source: PLoS Genet. 2012 Feb 2;8(2):e1002488. doi: 10.1371/journal.pgen.1002488 (PMC3271059; doi:10.1371/journal.pgen.1002488)
Supplement: Table S2 — List of deficiencies identified to cause semi-lethality. (PDF) [file pgen.1002488.s010.pdf]

Table S2. List of deficiencies identified to cause semi-lethality

Deficiency	Candidate region	Confirmed semi-lethality
Df(1)64c18	2E1-2;3C2	SL
Df(1)JC19	2F6;3C5	SL
Df(1)N-8	3C2-3;3E3-4	SL
Df(1)dm75e19	3C11;3E4	SL
Df(1)N73	5C2;5D5-6	SL
Df(1)g	12A3-10;12E9	SL
Df(1)RR79	16C;16F	SL
Df(3L)AC1	67A2;67D11-13	SL
Df(4)ED6366	102A1;102A6	SL
