# Supplementary material for: The Mitochondrial Chaperone Protein TRAP1 Mitigates α-Synuclein Toxicity
Source: PLoS Genet. 2012 Feb 2;8(2):e1002488. doi: 10.1371/journal.pgen.1002488 (PMC3271059; doi:10.1371/journal.pgen.1002488)
Supplement: Table S3 — Detailed summary of analyzed genes located within deficiencies causing a haploinsufficiency in combination with ddc>A53T. (PDF) [file pgen.1002488.s011.pdf]

Table S3. Detailed summary of analyzed genes located within deficiencies causing a haploinsufficiency in combination with *ddc>A53T*

**Deficiency *Df(1)dx81* (5C3-10;6C3-12)**				
**Cytological location**	**GENE NUMBER/ NAME**	**CG NUMBER**	**Vienna RNAi**	**BL Stock**
5C2-5C3	*CG15765*	*CG15765*		18597
5C3-5C3	*CG11462*	*CG11462*		
5C3-5C3	*CG12729*	*CG12729*		
5C5-5C5	*CG15764*	*CG15764*		
5C5-5C5	*CG3033*	*CG3033*	7086	
5C5-5C5	*mof*	*CG3025*		
5C5-5C6	*CG3016*	*CG3016*	7090	
5C6-5C6	*CG16721*	*CG16721*		
5C7-5C7	*Act5C*	*CG4027*	7139	
5C7-5C7	*CG4020*	*CG4020*		17170
5C7-5C7	*CG12236*	*CG12236*		
5C7-5C7	*CG3011*	*CG3011*	19208	
5C7-5C10	*CG3726*	*CG3726*		19187
5C10-5C10	*CG12728*	*CG12728*		
5C10-5C10	*CG32756*	*CG32756*		
5C10-5C10	*CG6041*	*CG6041*		
5C10-5C10	*CG32755*	*CG32755*		
5C10-5C10	*CG6048*	*CG6048*		
5D1-5D1	*CG6067*	*CG6067*		
5D1-5D1	*fs(1)M3*	*CG4790*		
5D1-5D1	*Grip*	*CG14447*		11239
5D1-5D1	*CG5966*	*CG5966*	13164	
5D1-5D2	*CG4766*	*CG4766*		
5D2-5D2	*mab-21*	*CG4746*		18541
5D2-5D2	*rux*	*CG4336*		17867
5D2-5D2	*CG5941*	*CG5941*		
5D2-5D3	*CG5937*	*CG5937*		
5D3-5D3	*CG5921*	*CG5921*	37875	
5D3-5D3	*CG5928*	*CG5928*		
5D3-5D3	*Tsp5D*	*CG4690*	45740	10093
5D3-5D3	*CG4666*	*CG4666*	36293	12674
5D4-5D4	*CG4660*	*CG4660*		
5D4-5D4	*Mipp2*	*CG4317*	14163	14247
5D4-5D4	*raptor*	*CG4320*	13112	
5D4-5D5	*Nep1*	*CG5905*	7108	15146
5D5-5D6	*Ca-alpha1T*	*CG15899*		19179
5D8-5D8	*CG32750*	*CG32750*		
5D8-5D8	*CG32751*	*CG32751*		

5E1-5E1	*vanin-like*	*CG32754*	50591	
5E1-5E1	*CG3599*	*CG3599*	16665	
5E1-5E1	*CG3774*	*CG3774*	30238	
5E1-5E1	*sqh*	*CG3595*	7916	
5E1-5E1	*CG14446*	*CG14446*	10274	
5E1-5E1	*CG14445*	*CG14445*		
5E1-5E3	*CG3585*	*CG3585*		
5E3-5E3	*Spx*	*CG3595*		
5E3-5E4	*Lag1*	*CG3576*		13138
5E3-5E3	*CG3781*	*CG3781*	7113	
5E4-5E4	*Spt6*	*CG3780*	40471	
5E4-5E4	*CG3566*	*CG3566*		
5E4-5E4	*Ubi-p5E*	*CG32744*		11854
5E4-5E4	*CR32745*	*CR32745*		
5E5-5E5	*CR11700*	*CR11700*		
5E5-5E5	*Top3beta*	*CG3458*	30627	17226
5E5-5E5	*wuho*	*CG15897*	41618	
5E5-5E5	*Rpt4*	*CG3455*	49574	12131
5E5-5E6	*CG15896*	*CG15896*		
5E6-5E6	*CG15892*	*CG15892*		
5E6-5E6	*CG15891*	*CG15891*		
5E6-5E7	*CG3815*	*CG3815*		
5E7-5E7	*CG12219*	*CG12219*		
5E7-5E8	*CG3823*	*CG3823*		
5F1-5F1	*CG15894*	*CG15894*		18304
5F2-5F2	*CG3842*	*CG3842*	7117	19166
5F2-5F2	*CG3847*	*CG3847*		
5F2-5F2	*CG3446*	*CG3446*	42696	
**5F2-5F4**	***kdn***	***CG3861***	**26301**	**14436**
5F4-5F4	*swa*	*CR32747*		18431
5F4-5F4	*Marf*	*CG3429*		
5F4-5F4	*PpV*	*CG12217*	31690	
5F5-5F5	*CG33664*	*CG33664*		
5F6-5F6	*CG33669*	*CG33669*		
6A1-6A1	*CG33668*	*CG33668*		
6A2-6A2	*CG3367*	*CG3367*		18325
6A2-6A2	*CG33667*	*CG33667*		
6A2-6A2	*CG33666*	*CG33666*		
6A2-6A2	*CG33665*	*CG33665*	51606	
6A3-6A3	*CG12543*	*CG12543*		
6B1-6B1	*tRNA:CR32748-RA*			
6B1-6B1	*tRNA:CR32748*			
6B1-6B1	*CG3918*	*CG3918*		19185
6B1-6B1	*CG3342*	*CG3342*		18549
6B1-6B1	*Spat*	*CG3926*		
6B1-6B1	*RpL7A*	*CG3314*	43760	18043

6B1-6B1	*snoRNA:Or-CD10-RA*			
6B1-6B1	*snoRNA:Or-CD10*			
6B1-6B2	*dx*	*CG3929*	7795	
6B2-6B3	*CG34417*	*CG34417*		13234
6C1-6C1	*CG17717*	*CG17717*		
6C1-6C1	*Pat1*	*CG10695*	27307	18059
6C1-6C1	*APC7*	*CG14444*	17261	15885
6C1-6C3	*CG3973*	*CG3973*	34772	12446
6C2-6C2	*CG14443*	*CG14443*		
6C3-6C3	*CG3226*	*CG3226*	47034	
6C3-6C3	*l(1)G0148*	*CG32742*		
6C3-6C4	*CG3224*	*CG3224*	29561	11243
6C4-6C4	*Ctr1A*	*CG3977*	46757	11243
6C4-6C4	*CG3198*	*CG3198*		15397
6C4-6C5	*Mcm6*	*CG4039*	13661	
6C5-6C5	*CG3192*	*CG3192*	30413	
6C5-6C5	*l(1)G0255*	*CG4094*	34797	
6C5-6C5	*CG4095*	*CG4095*	47685	
6C6-6C6	*Pink1*	*CG4523*	21860	
6C6-6C6	*CG3184*	*CG3184*	25880	
6C6-6C7	*CG14442*	*CG14442*		18974
6C7-6C7	*CG32741*	*CG32741*		
6C7-6C7	*CG14440*	*CG14440*	44474	12468
6C9-6C9	*CG14441*	*CG14441*		19335
6C9-6C10	*CG3168*	*CG3168*	48010	12731
6C10-6C10	*RpL17*	*CG3203*	41778	10994
6C10-6C10	*snoRNA:Psi28S-3436b*			
6C10-6C10	*snoRNA:Psi28S-3436b-RA*			
6C10-6C10	*snoRNA:Psi28S-3436a-RA*			
6C10-6C10	*snoRNA:Psi28S-3436a*			
6C10-6C10	*snoRNA:Psi28S-1060-RA*			
6C10-6C10	*snoRNA:Psi28S-1060*			
6C10-6C10	*snoRNA:Psi28S-1232*			
6C10-6C10	*snoRNA:Psi28S-1232-RA*			
6C10-6C10	*tRNA:CR32740*			
6C10-6C10	*tRNA:CR32740-RA*			
6C11-6C12	*CG14439*	*CG14439*		
6C12-6C13	*CG14438*	*CG14438*		13068

Deficiency *Df(1)BK10* (15F2-16C5)

Cytological location	GENE NUMBER/ NAME	CG NUMBER	Vienna RNAi	BL Stock
15F2-15F2	*CG8918*	*CG8918*	28994	
15F3-15F3	*CG5070*	*CG5070*	6150	
15F3-15F3	*CG32564*	*CG32564*		

15F3-15F3	*CG32563*	*CG32563*		
15F3-15F3	*CG12995*	*CG12995*		
15F3-15F4	*CG18258*	*CG18258*	20621	
15F4-15F4	*CG5162*	*CG5162*		
15F4-15F4	*CG12998*	*CG12998*		
15F4-15F4	*CG5172*	*CG5172*		
15F4-15F4	*CG12997*	*CG12997*		
15F4-15F4	*CG34327*	*CG34327*		
15F4-15F4	*CG10598*	*CG10598*		
15F4-15F4	*CG10597*	*CG10597*	6157	
15F4-15F4	*CG8915*	*CG8915*	28857	
15F4-15F4	*CG8675*	*CG8675*	26997	
15F4-15F4	*CG12996*	*CG12996*		
15F4-15F7	*f*	*CG42864*	33200	
15F7-15F7	*CG8664*	*CG8664*	47568	
15F7-15F7	*CG8661*	*CG8661*		
15F8-16A1	*Fim*	*CG86401*	47514	19171
16A1-16A1	*CG5445*	*CG5445*		14274
16A1-16A1	*B-H2*	*CG5488*		11437
16A4-16A5	*B-H1*	*CG5529*		
16A5-16A5	*CG8611*	*CG8611*	28936	
16A5-16A5	*CG12994*	*CG12994*	11864	
16A5-16A5	*CG5613*	*CG5613*	24694	18602
16A5-16A5	*CG12993*	*CG12993*	20304	
16A5-16A5	*CG8568*	*CG8568*		
16A5-16B1	*CG8557*	*CG8557*	28927	18715
16B3-16B3	*CG12432*	*CG12432*		
16B4-16B4	*ppk23*	*CG8527*	39580	
16B5-16B6	*l(1)G0222*		24107	
16B6-16B7	*CG12991*	*CG12991*		10234
16B6-16B6	*CG12992*	*CG12992*		
16B7-16B7	*X11L*	*CG5675*	27479	18147
16B9-16B9	*CG8408*	*CG8408*	12432	
16B9-16B10	*CG8326*	*CG8326*	23760	
16B10-16B10	*CG8316*	*CG8316*		19865
16B10-16B10	*CG5703*	*CG5703*	10140	
16B10-16B10	*CG8289*	*CG8289*	24279	19336
16B10-16B10	*CG5800*	*CG5800*	27519	
16B10-16B10	*RhoGAPp190*	*CG32555*		20177
16B10-16B10	*CG8211*	*CG8211*	24329	12545
16B10-16B12	*beta-Spec*	*CG5870*	42083	13310
16B12-16B12	*CG12990*	*CG12990*	6160	
16B12-16C1	*CG32556*	*CG32556*		
16C1-16C1	*CG8188*	*CG8188*		11439
16C1-16C1	*par-6*	*CG5884*	19730	
16C1-16C1	*CG8173*	*CG8173*	35845	

16C1-16C8	*CG32560*	*CG32560*	20983	12700
16C5-16C5	*CG32559*			
16C5-16C6	*CG15816*	*CG15816*	19616	

Deficiency *Df(2L)Exel6011* (25C7-25D5)

Cytological location	GENE NUMBER/ NAME	CG NUMBER	Vienna RNAi	BL Stock
25C7-25C10	*Msp-300*	*CG33715*		13707
25C9-25C9	*snoRNA:Psi28S-2263*			
25C9-25C9	*snoRNA:Psi28S-2263-RA*			
25C9-25C9	*snmRNA:765*			
25C9-25C9	*snmRNA:765-RA*			
25C10-25C10	*snmRNA:128*			
25C10-25C10	*snmRNA:128-RA*			
25C10-25C10	*snoRNA:Psi18S-525k-RA*			
25C10-25C10	*snoRNA:Psi18S-525k*			
25C10-25C10	*snmRNA:158*			
25C10-25C10	*snmRNA:158-RA*			
25C10-25C10	*Cyp28d2*	*CG6081*	7868	
25C10-25C10	*Cyp28d1*	*CG10833*	7870	
25C10-25D1	*CG7742*	*CG7742*	25535	
25D1-25D1	*CG14034*	*CG14034*	25536	
25D1-25D1	*TpnC25D*	*CG6514*	27649	
25D1-25D2	*tkv*	*CG14026*	3059	
25D2-25D2	*tRNA:CR31971*			
25D2-25D2	*tRNA:CR31971-RA*			
25D2-25D2	*tRNA:CR31914*			
25D2-25D2	*tRNA:CR31914-RA*			
25D2-25D2	*CG14033*	*CG14033*		14403
25D2-25D2	*Cyp4ac1*	*CG14032*		
25D2-25D2	*Cyp4ac2*	*CG17970*	16987	
25D2-25D2	*Cyp4ac3*	*CG14031*	48920	
25D2-25D3	*Bsg25D*	*CG14025*	13398	
25D3-25D3	*Bub1*	*CG14030*	24833	
25D4-25D5	*vri*	*CG14029*	5650	
25D5-25D5	*CG14024*	*CG14024*	13402	
25D5-25D6	*CG14023*	*CG14023*	36480	

Deficiency *Df(3L)h-i22* (66D10-66D12)

Cytological location	GENE NUMBER/ NAME	CG NUMBER	Vienna RNAi	BL Stock
66D10-66D10	*CG6486*	*CG6486*	43804	
66D10-66D10	*h*	*CG6494*		11730
66D11-66D11	*SrpRbeta*	*CG33162*	5150	12072
66D11-66D11	*CG32022*	*CG32022*	10105	

66D11-66D12	*CG6511*	*CG6511*		
66D12-66D12	*Cp18*	*CG6517*	39622	
66D12-66D12	*Cp15*	*CG6519*	13860	
66D12-66D12	*Cp19*	*CG6524*	33286	
66D12-66D12	*Cp16*	*CG6533*	19870	
66D12-66D12	*Prm*	*CG5939*		11752
66D12-66D12	*CG13306*	*CG13306*	44960	
66D12-66D12	*CG6576*	*CG6576*	26695	
66D12-66D15	*Fhos*	*CG32030*		16022
66D14-66D14	From CG5804 in overlap def BL 8702- not lethal	*CG5804*	*23587*	
66D15-66D15	*CG13310*	*CG13310*	43960	
66D15-66D15	*CG13311*	*CG13311*	51574	
66D15-66D15	*CG34426*	*CG34426*		
66D15-66D15	*CG32023*	*CG32023*		
66D15-66D15	*CG34427*	*CG34427*		
66D15-66D15	*CG13312*	*CG13312*		
66D15-66D15	*CG32024*	*CG32024*		
66D15-66D15	*CG13308*	*CG13308*		
66D15-66D15	*CG13309*	*CG13309*		
66E1-66E3	*dally*	*CG4974*		
66E1-66E1	*CG32026*	*CG32026*		
**Deficiency *Df(3R)e-R1* (93B7-93D3)**				
**Cytological location**	**GENE NUMBER/ NAME**	**CG NUMBER**	**Vienna RNAi**	**BL Stock**
93B5-93B7	*Dhc93AB*	*CG3723*	41947	
93B7-93B7	*CG12278*	*CG12278*	29484	
93B7-93B7	*CG31189*	*CG31189*	22962	
93B7-93B7	*CG31207*	*CG31207*		
93B7-93B7	*CG7079*	*CG7079*		
93B7-93B7	*CG17279*	*CG17279*		
93B7-93B8	*Mvl*	*CG3671*	44000	19886
93B8-93B9	*Cortactin*	*CG3637*		22381
93B9-93B10	*AnnIX*	*CG5730*	27493	
93B10-93B10	*r-l*	*CG3593*	21688	17982
93B10-93B10	*dmrt93B*	*CG5737*	41048	
93B10-93B10	*CG7056*	*CG7056*		17943
93B10-93B11	*RhoGAP93B*	*CG3421*	41934	19720
93B11-93B12	*CG7044*	*CG7044*	27811	12413
93B12-93B12	*CG5745*	*CG5745*	35034	
93B12-93B12	*sec15*	*CG7034*		
93B12-93B12	*rtet*	*CG5760*	44002	11714
93B12-93B13	*Rab11*	*CG5771*	22198	12418
93B13-93B13	*ppan*	*CG5786*	39001	11557

93B13-93B13	*CG17282*	*CG17282*		
93B13-93C1	*slmb*	*CG3412*		11493
93C1-93C1	*CG5793*	*CG5793*	47883	16067
93C1-93C1	*Obp93a*	*CG17284*	2558	
93C1-93C1	*CG10825*	*CG10825*	31360	
93C1-93C1	*CG7009*	*CG7009*	27790	18008
93C1-93C1	*CG5798*	*CG5798*	8931	
93C1-93C1	*CG5802*	*CG5802*	6801	13460
93C1-93C5	*SNF4Agamma*	*CG17299*		13088
93C2-93C2	*CG10824*	*CG10824*	16588	
93C2-93C2	*CG5810*	*CG5810*	44988	
93C2-93C2	*CG7000*	*CG7000*	42496	
93C6-93C6	*CG5862*	*CG5862*	45155	
93C6-93C6	*CG3353*	*CG3353*	41920	7353
93C6-93C7	*CG5871*	*CG5871*	41822	13618
93C7-93C7	*CG3337*	*CG3337*	29170	
93C7-93C7	*NELF-A*	*CG5874*	43211	15189
93C7-93D1	*e*	*CG3331*	45689	
93D1-93D1	*CG5892*	*CG5892*	6807	18496
93D1-93D2	*ETHR*	*CG5911*	42716	
93D2-93D2	*Rab1*	*CG3320*		17936
93D2-93D2	*AP-2sigma*	*CG6056*	34148	13478
93D2-93D2	*CG5919*	*CG5919*	28402	
93D2-93D2	*CG3308*	*CG3308*	43756	
93D2-93D2	*CG3301*	*CG3301*	43255	18137
93D2-93D4	*SIFR*	*CG10823*	1783	16202
93D3-93D3	*CG17298*	*CG17298*		
93D4-93D4	*Hsromega-RB*			
93D4-93D4	*Hsromega*			
93D4-93D4	*Hsromega-RA*			
93D4-93D4	*Hsromega-RC*			
