# Supplementary material for: The Mitochondrial Chaperone Protein TRAP1 Mitigates α-Synuclein Toxicity
Source: PLoS Genet. 2012 Feb 2;8(2):e1002488. doi: 10.1371/journal.pgen.1002488 (PMC3271059; doi:10.1371/journal.pgen.1002488)
Supplement: Table S4 — Deficiencies causing a decline in DA. (PDF) [file pgen.1002488.s012.pdf]

Table S4. Deficiencies causing a decline in DA

Deficiency	Candidate region	Head DA (4 week as % 1 week)
Df(1)ct-J4	7A2-3;7C1	26.82
Df(1)4b18	14B8;14C1	47.44
Df(2L)ast2	21D1-2;22B23	47.06
Df(2L)spd[j2]	27C1-2;28A	47.48
Df(2L)Mdh	30D-30F;31F	29.62
Df(2L)BSC32	32A1-2;32C5-D1	31.34
Df(2L)BSC147	34C1;34C6	27.51
Df(2R)nap9	42A1-2;42E6-F1	19.81
Df(2R)H3E1	44D1-4;44F12	49.94
Df(2R)w45-30n	45A6-7;45E2-3	47.2
Df(2R)BSC161	54B2;54B17	21.48
Df(2R)BSC45	54C8-D1;54E2-7	46.81
Df(2R)P34	55E2-4;56C1-11	49.94
Df(2R)59AD	59A1-3;59D1-4	41.00
Df(2R)vir130	59B;59D8-E1	39.85
Df(3L)ZN47	64C;65C	40.69
Df(3L)XDI98	65A2;65E1	24.71
Df(3L)BSC33	65E10-F1;65F2-6	41.51
Df(3L)vin7	68C8-11;69B4-5	26.11
Df(3R)Scr	84A1-2;84B1-2	29.55
Df(3R)p-XT103	85A2;85C1-2	46.60
Df(3R)by10	85D8-12;85E7-F1	45.14
Df(3R)BSC137	94F1;95A4	48.79
C(4)RM	101F1;102F8	33.43
