# Supplementary material for: The Mitochondrial Chaperone Protein TRAP1 Mitigates α-Synuclein Toxicity
Source: PLoS Genet. 2012 Feb 2;8(2):e1002488. doi: 10.1371/journal.pgen.1002488 (PMC3271059; doi:10.1371/journal.pgen.1002488)
Supplement: Table S5 — Primers. (PDF) [file pgen.1002488.s013.pdf]

Table S5. Primers

Fly β-Actin5c	for: 5′-ccagtcattcctttcaaacc-3
	rev: 5′-gcaacttcttcgtcacacatt-3
Fly TRAP1	for: 5′-aggcagagtcaccgatcc-3′
	rev: 5′-tgatgcctgcttggtctc-3′
Human 18S	Quantitect primers (Qiagen, Germany)
hTRAP1	for: 5’-cagaccaatgccgagaaag-3’
	rev: 5’-caccagctcttcctgtgtca-3’
hTRAP1-*Bgl*II	for: 5’-gaagatctatggcgcgcgagctgcgggcgctgctgc-3’
hTRAP1-*Xho*I	rev: 5’-ccgctcgagtcagtgtcgctccagggccttgaca-3’
TRAP1[D158N]	for: 5´- ggcaccatcaccatccagaatactggtatcggg-3`
	rev: 5´- cccgataccagtattctggatggtgatggtgcc-3`
