# Supplementary material for: The Mitochondrial Chaperone Protein TRAP1 Mitigates α-Synuclein Toxicity
Source: PLoS Genet. 2012 Feb 2;8(2):e1002488. doi: 10.1371/journal.pgen.1002488 (PMC3271059; doi:10.1371/journal.pgen.1002488)
Supplement: Text S1 — Brief summary of screen results. (DOC) [file pgen.1002488.s014.doc]

**Detailed information on *Drosophila* Screen**

To screen for modifications of [A53T]α-Synuclein neurotoxicity, we used loss of DA over time as the read-out. Flies from the “Bloomington Deficiency Kit” (270 lines, each containing a defined chromosomal deletion) were crossed to *ddc>A53T* flies. In the F1 generation, flies with *ddc>A53T* and the respective chromosomal deletion were collected and DA content in heads was determined at 1 and 4 weeks of age. An overall summary of the primary data is shown in the tables below. Results of the initial screen were categorized in two groups on the basis of

1.) lethality (Table S1) and reduced viability (offspring did not live until the 4-week collection point or crosses were without viable offspring, Table S2), or

2.) low DA levels (less than 50% DA at 4-week time point).

To confirm the results of the initial screen, candidates from the primary screen were analyzed at least twice. In group #1, five candidate deficiencies induced a synthetic haploinsufficiency in combination with *ddc>A53T* and nine candidates were semi-lethal. 24 lines were categorized as group #2.

Detailed analysis of group #1: For the deficiencies identified to cause synthetic haploinsufficiencies (Table S1), we tried to assay all candidate genes (RNAi and/or mutant alleles if available) within the deficiency. Accordingly, *ddc>A53T* flies were crossed to mutant alleles/RNAi lines of candidate genes and presence of viable transheterozygous offspring served as readout. As we model a human neurodegenerative disease in flies, our main focus was on fly genes having a human homolog. A summary of this analysis is shown in Table S3.

In case of a *kdn[KG04873]* (loss-of-function allele), we were unable to detect transheterozygous flies (*kdn[KG04873]/+;;ddc>A53T/+*). All other transheterozygous combinations (either UAS-RNAi directed against indicated genes or mutant alleles) were viable.

In case of *Df(1)dx81*, analysis was stopped after the identification of a *kdn* allele (*kdn^KG04873^*) to cause lethality in combination with *ddc>A53T*. The remaining candidate deficiencies are still subject of analysis. However, we are limited according to availability of mutant alleles and RNAi lines. In the case of the present screen it is important to keep in mind that there is a high chance to retrieve candidate deficiencies where the observed phenotype is caused by multiple gene deletions. Therefore, the effect induced by the deficiencies cannot be recapitulated by a single gene mutation. For a detailed list of genes analyzed using Vienna RNAi and/or Bloomington mutant allele fly lines see below.

Detailed analysis of group #2: Analysis of the candidate deficiencies, causing a decline in DA content of *ddc>A53T* flies, is still ongoing. Further analysis of the candidate deficiencies will hopefully lead to the identification of other genetic interactors of [A53T]α-Synuclein-induced DA decline. The degree of DA decline and a complete list of these deficiencies is displayed in Table S4.

Given the identification of *kdn*, coding for citrate synthase – the pace-making enzyme of the Krebs cycle - mitochondrial gene products became a focus of our analysis. Thus, we switched from a random to a biased approach. The mitochondrial protein TRAP1 is known as downstream target of PINK1 and is located in the deleted area of the deficiency *Df(2R)nap9* found to cause the highest decline of DA levels in *ddc>A53T* flies. Therefore, TRAP1 was chosen for detailed analysis. In addition, we cross-referenced our screening results with known modulators of mitochondrial function (Chen *et al.*, 2008). Of all genes analyzed approx. 38 % enhanced [A53T]α-Synuclein-induced DA decline. Further analysis of these genes and their impact on [A53T]α-Synuclein-induced toxicity will contribute to the identification of new pathways and to the understanding of the mechanisms underlying PD pathology.
